# A Non-imaging High Throughput Approach to Chemical Library Screening at the Unmodified Adenosine-A_3_ Receptor in Living Cells

**DOI:** 10.3389/fphar.2017.00908

**Published:** 2017-12-13

**Authors:** Maria Augusta Arruda, Leigh A. Stoddart, Karolina Gherbi, Stephen J. Briddon, Barrie Kellam, Stephen J. Hill

**Affiliations:** ^1^Division of Physiology, Pharmacology and Neuroscience, School of Life Sciences, Medical School, Queen's Medical Centre, University of Nottingham, Nottingham, United Kingdom; ^2^Centre of Membrane Proteins and Receptors, University of Birmingham and University of Nottingham, The Midlands, United Kingdom; ^3^Vice-Diretoria de Ensino, Pesquisa e Inovacao, Farmanguinhos, Fiocruz, Rio de Janeiro, Brazil; ^4^Centre for Biomolecular Sciences, School of Pharmacy, University of Nottingham, Nottingham, United Kingdom

**Keywords:** adenosine receptors, fluorescent ligands, adenosine A_3_ receptor, high throughput screening, LOPAC library

## Abstract

Recent advances in fluorescent ligand technology have enabled the study of G protein-coupled receptors in their native environment without the need for genetic modification such as addition of N-terminal fluorescent or bioluminescent tags. Here, we have used a non-imaging plate reader (PHERAstar FS) to monitor the binding of fluorescent ligands to the human adenosine-A_3_ receptor (A_3_AR; CA200645 and AV039), stably expressed in CHO-K1 cells. To verify that this method was suitable for the study of other GPCRs, assays at the human adenosine-A_1_ receptor, and β_1_ and β_2_ adrenoceptors (β_1_AR and β_2_AR; BODIPY-TMR-CGP-12177) were also carried out. Affinity values determined for the binding of the fluorescent ligands CA200645 and AV039 to A_3_AR for a range of classical adenosine receptor antagonists were consistent with A_3_AR pharmacology and correlated well (*R*^2^ = 0.94) with equivalent data obtained using a confocal imaging plate reader (ImageXpress Ultra). The binding of BODIPY-TMR-CGP-12177 to the β_1_AR was potently inhibited by low concentrations of the β_1_-selective antagonist CGP 20712A (pK_i_ 9.68) but not by the β_2_-selective antagonist ICI 118551(pK_i_ 7.40). Furthermore, in experiments conducted in CHO K1 cells expressing the β_2_AR this affinity order was reversed with ICI 118551 showing the highest affinity (pK_i_ 8.73) and CGP20712A (pK_i_ 5.68) the lowest affinity. To determine whether the faster data acquisition of the non-imaging plate reader (~3 min per 96-well plate) was suitable for high throughput screening (HTS), we screened the LOPAC library for inhibitors of the binding of CA200645 to the A_3_AR. From the initial 1,263 compounds evaluated, 67 hits (defined as those that inhibited the total binding of 25 nM CA200645 by ≥40%) were identified. All compounds within the library that had medium to high affinity for the A_3_AR (pK_i_ ≥6) were successfully identified. We found three novel compounds in the library that displayed unexpected sub-micromolar affinity for the A_3_AR. These were K114 (pK_i_ 6.43), retinoic acid *p*-hydroxyanilide (pK_i_ 6.13) and SU 6556 (pK_i_ 6.17). Molecular docking of these latter three LOPAC library members provided a plausible set of binding poses within the vicinity of the established orthosteric A_3_AR binding pocket. A plate reader based library screening using an untagged receptor is therefore possible using fluorescent ligand opening the possibility of its use in compound screening at natively expressed receptors.

## Introduction

G protein-coupled receptors (GPCRs) represent the largest family of cell surface receptors and account for approximately 4% of the entire protein-coding human genome. There are approximately 700 separate GPCRs of which over 300 are non-olfactory receptors (Kuder and Kieć-Kononowicz, 2014). Based on sequence homology, five distinct families of non-olfactory receptors have been proposed: Family A/Rhodopsin, Family B/secretin, Adhesion GPCRs, Family C/Glutamate, and Family F/frizzled (Guo et al., 2012). Family A contains the largest number of the non-olfactory GPCRs including many of the most widely studied receptors, each of which acts to translate extracellular signals into intracellular effects by activating both heterotrimeric G protein-dependent and -independent signaling cascades (Castro et al., 2005; Guo et al., 2012). Importantly, these family A GPCRs are also currently targeted by a large number of clinically used drugs and are validated targets for a significant number of drug discovery programmes.

Adenosine is one biological transmitter which plays a vital homeostatic role and acts via a family of Class A GPCRs comprising four distinct subtypes: namely the adenosine-A_1_ receptor (A_1_AR), A_2A_AR, A_2B_AR, and A_3_AR (Fredholm et al., 2011). Both the A_1_AR and A_3_ARs inhibit intracellular cAMP formation by activating inhibitory G_i_ proteins, whilst the A_2A_AR and A_2B_ARs generally stimulate cAMP formation via stimulatory G_s_ proteins. Adenosine-mediated signaling has been implicated in a number of pathological states. For instance, the signaling pathways regulated by these receptors can promote angiogenesis (Headrick et al., 2013) and reduce inflammation (Antonioli et al., 2014). Within this family, the A_3_AR is a promising molecular target for the control of a range of pathological conditions including cancer (Montinaro et al., 2013; Nakamura et al., 2015; Cao et al., 2017; Joshaghani et al., 2017), inflammation (Cohen et al., 2014; Yoshida et al., 2017), autoimmune diseases (Ravani et al., 2017), ischaemia (Mulloy et al., 2013; González-Fernández et al., 2014; Hussain et al., 2014; Ohana et al., 2016) and chronic neuropathic pain (Little et al., 2015; Tosh et al., 2015), making it an important target for drug development (Borea et al., 2015). As a consequence, identifying new screening methods for discovery of novel chemical scaffolds which bind to the A_3_AR would be beneficial.

With this in mind, it is of note that recent advances in fluorescent ligand technology have enabled unlabeled GPCRs to be studied in their native environment without any need for genetic modification through the addition of a bioluminescent or fluorescent tag. For instance fluorescent ligands have been used to study various aspects of GPCR pharmacology including ligand binding, receptor-ligand kinetics, receptor localization and trafficking (Stoddart et al., 2015b). Of particular relevance to purinergic drug discovery, Stoddart et al. (2012) developed a competitive binding assay for the human A_3_AR and A_1_AR in live cells, using a high content screening (HCS) platform that allowed the screening of small fragment libraries. This assay system was also used to validate the pharmacology of A_3_AR selective compounds that were identified from virtual screening of homology models (Ranganathan et al., 2015). However, a disadvantage of this technique is that it involves the acquisition and analysis of a large number of images which can impose severe time, data handling and storage limitations at the early stages of drug discovery, particularly in hit discovery, when very large libraries (>100,000 compounds) are used in initial screening campaigns (Tomasch et al., 2012). In this work, we show that such a competitive fluorescent based binding screen is possible on a higher throughput, non-imaging-based platform using two structurally unrelated fluorescent antagonists. The suitability of this assay for higher throughput screens has been demonstrated by screening a library of pharmacological active compounds (LOPAC) against the native human A_3_AR in living cells, with a view to identifying potential novel scaffolds for A_3_AR ligands.

## Results

### Comparison of high content (HCS) and high throughput (HTS) screening platforms for measuring competition binding to the A_3_AR

**Figure 1:**
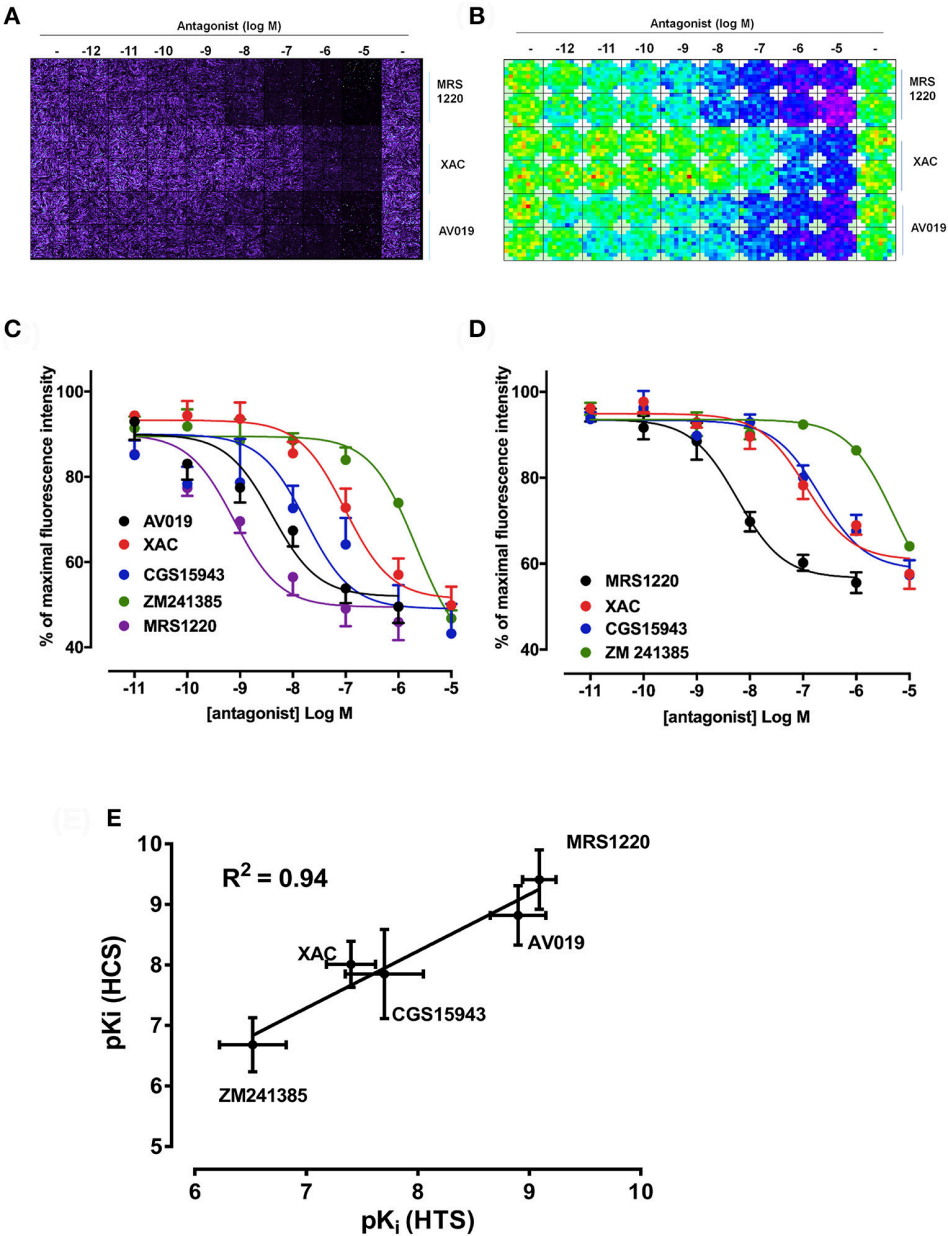
Competition binding at the A_3_AR using fluorescent ligands. CHO cells expressing the A_3_AR were incubated with 25 nM CA200645 and increasing concentrations of MRS1220, XAC, or AV019. **(A)** Four images per well were obtained on the IX Ultra confocal plate reader and resulting images shown as a montage. **(B)** Montage fluorescence intensity measurement of the same plate obtained using the FS PHERAstar where blue, green, yellow and red pixels represents increasing intensity of fluorescence. **(C)** Competition curves at the A_3_AR generated from the total fluorescence intensity measured on the PHERAstar FS microplate reader for five adenosine receptor antagonists. **(D)** CHO A_3_AR cells were incubated with increasing concentrations of antagonist and 5 nM AV039 for 1 h, 37°C, washed and fluorescence intensity assessed using the PHERAstar FS. **(E)** Correlation between pKi values obtained using the IX Ultra (high content screening; HCS) and the PHERAstar FS (high throughput screening; HTS) for the data obtained using CA200645 as fluorescent ligand. Data were normalized to the maximal intensity observed per experiment and each data point represents the mean ± SEM from *n* number of experiments (See Table 1) performed in triplicate.

As previously described, competition binding assays have been performed on cells expressing the wild type human A_3_AR using the fluorescent adenosine receptor antagonist CA200645 by automated image acquisition using an ImageXpress (IX) Ultra confocal imaging plate reader (Stoddart et al., 2012). In order to see if this method could be translated into a faster non-imaging format, we directly compared HCS and plate reader based CA200645 binding by sequentially reading the same samples on the PHERAstar FS (BMG technologies) then the IX Ultra. As shown in the IX Ultra plate image in Figure 1A, binding of 25 nM CA200645 was clearly detected, and was subsequently displaced by increasing concentrations of competing (unlabeled) antagonists. The same 96-well plate was also measured on a standard non-imaging fluorescence plate reader (PHERAstar FS), with 81 separate repeat reads per well to take into account variation in cell density, and a similar pattern of fluorescence was observed (Figure 1B). The montage images from both instruments show that the high affinity A_3_AR antagonist MRS1220, AV019 (compound 1 in Vernall et al., 2012) and the non-selective adenosine receptor antagonist xanthine amine congener (XAC) caused a concentration-dependent reduction in the fluorescence intensity observed with 25 nM CA200645 alone. Competition binding curves were generated from the quantified data (Figure 1C), and pK_i_ values for the five adenosine receptor antagonists obtained, which were comparable to values reported in the literature (Table 1). Comparison of the affinity values from the HTS platform (PHERAstar) to those from the HCS platform (IX Ultra) showed a high degree of correlation (*R*^2^ = 0.94) (Figure 1E) and we have previously shown that affinity values obtained from the HCS platform correlated well with values obtained in a functional assay (Stoddart et al., 2012). In addition to the XAC based fluorescent ligand CA200645, a structurally distinct and highly selective fluorescent A_3_AR antagonist was also used (AV039; compound 19 in Vernall et al., 2012). As with CA200645, using 5 nM AV039 as label, competition binding experiments measured on the PHERAstar FS produced the expected rank order of antagonist affinity for the A_3_AR (Figure 1D, Table 1).

**Table 1:** Affinity of compounds measured at the A_1_AR and A_3_AR: Affinity values from the PHERAstar HTS assay for unlabeled ligands measured on CHO cells expressing the A_3_AR or the A_1_AR using 25 nM CA200645 or 5 nM AV039.

	**A**_**3**_**AR**				**Literature values**	**A**_**1**_**AR**		**Literature values**
	**CA200645**		**AV039**			**CA200645**		
	**pK_i_**	***n***	**pK_i_**	***n***		**pK_i_**	***n***	
MRS1220	9.30 ± 0.32	5	9.21 ± 0.12	6	9.02	7.35 ± 0.19	5	7.14
AV019	8.82 ± 0.28	4	ND	–	8.51	ND	–	5.93
XAC	8.06 ± 0.16	5	8.04 ± 0.22	4	7.85	7.70 ± 0.08	4	7.54
CGS15943	7.91 ± 0.20	3	7.91 ± 0.01	3	8.18	8.35 ± 0.16	3	8.95
ZM241385	6.63 ± 0.20	3	6.32 ± 0.28	3	6.74	6.54 ± 0.04	3	6.68

*Values represent mean ± SEM from n number of experiments performed in triplicate. ND, Not determined. Literature values for both A_3_AR and A_1_AR taken from Stoddart et al. (2012)*.

### Application to A_1_AR and β-adrenoceptors

**Figure 2:**
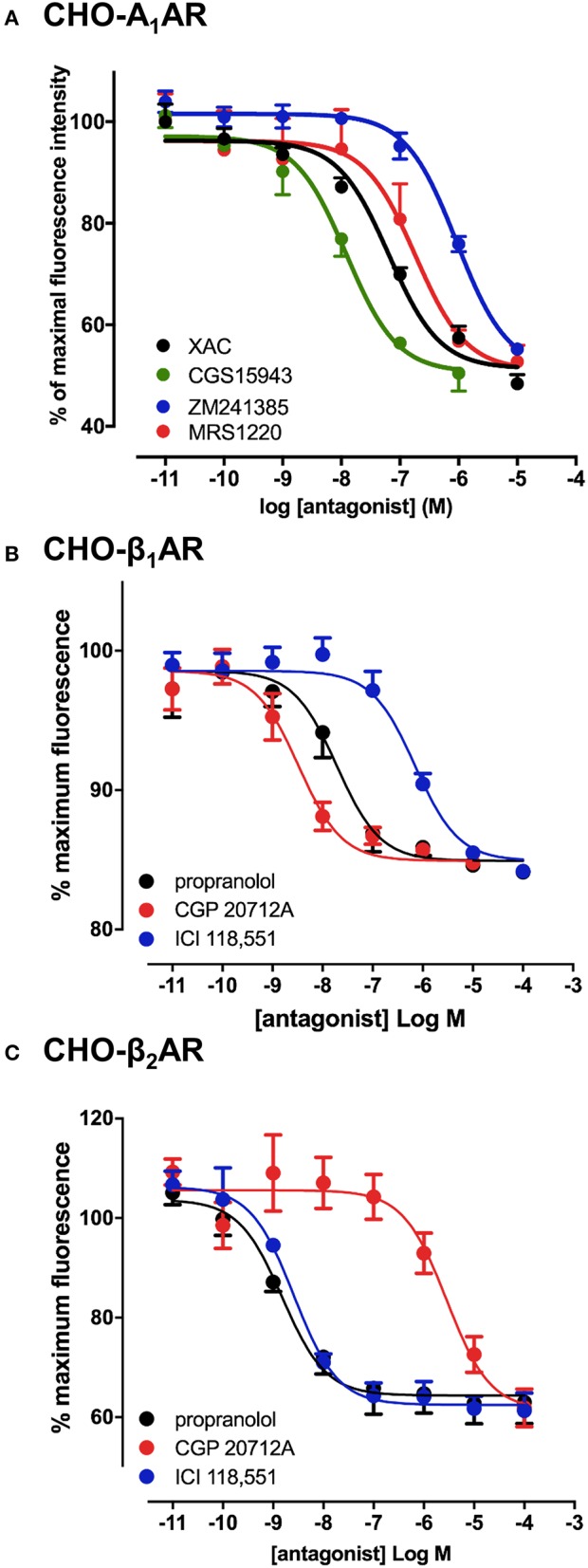
Competition binding assays at the adenosine A_1_ and β_1_/β_2_-adrenoceptors. CHO cell lines stably expressing A_1_AR **(A)**, β_1_AR **(B)** or the β_2_AR **(C)** were incubated with 25 nM CA200645 (A_1_AR) or 10 nM BODIPY-TMR-CGP (β_1_AR and β_2_AR), in the absence or the presence of increasing concentrations of antagonists. Fluorescence intensity in each well was monitored using the PHERAstar FS. Values are mean ± SEM from 3–6 independent experiments performed in triplicate.

To verify that the experimental approach used for the A_3_AR was suitable for the study of other GPCRs, we conducted the same experimental design with CA200645 on CHO cells expressing the human A_1_AR, since this fluorescent ligand also binds with high affinity to this receptor (Stoddart et al., 2012). This is important, since being able to screen for compound selectivity is an important aspect of developing a screening methodology. As with the A_3_AR, a clear concentration-dependent decrease in fluorescence intensity was detected on the HTS plate reader in the presence of four different adenosine receptor antagonists (Figure 2A). The affinity values from these data were consistent with A_1_AR pharmacology with CGS 15943 showing the highest affinity and MRS1220 exhibiting a lower affinity than at the A_3_AR. In addition, ZM241385, an A_2A_AR selective antagonist showed the expected low affinity at the A_1_AR (Table 1).

The confocal based fluorescent ligand binding assay has also been recently applied to study the pharmacology of the β_1_AR using BODIPY-TMR labeled CGP 12177 (BODIPY-TMR-CGP; Gherbi et al., 2014) and we therefore also tested whether ligand binding to the β_1_AR and β_2_AR could also be monitored using the HTS platform in order to develop a counter screen for the A_3_AR. As shown in Figure 2B, in CHO cells expressing either the β_1_AR or β_2_AR, binding of BODIPY-TMR-CGP could be clearly detected, and clear competition binding was observed with all three βAR ligands at both receptors. Importantly, the β_1_AR selective antagonist CGP 20712A displayed the highest affinity at the β_1_AR and the β_2_AR selective antagonist ICI 118551 the lowest (Table 2), whilst this rank order was reversed at the β_2_AR, with ICI 118551 showing the highest affinity and CGP20712A the lowest affinity (Figure 2C, Table 2).

**Table 2:** Affinity of compounds measured at the β_1_AR and β_2_AR: Affinity values for β-adrenoceptor ligands measured in CHO cells expressing the β_1_AR or the β_2_AR using 10 nM of BODIPY-TMR-CGP in the HTS format fluorescent ligand binding assay.

	**β**_**1**_**AR**		**β**_**2**_**AR**	
	**pK_i_**	***n***	**pK_i_**	***n***
Propranolol	8.89 ± 0.16	3	9.00 ± 0.09	3
CGP 20712A	9.68 ± 0.12	3	5.68 ± 0.06	3
ICI 118,551	7.40 ± 0.03	3	8.73 ± 0.07	3

*Values represent mean ± SEM from three experiments performed in triplicate*.

### Screening of a focused library of pharmacologically active ligands at the A_3_AR

**Figure 3:**
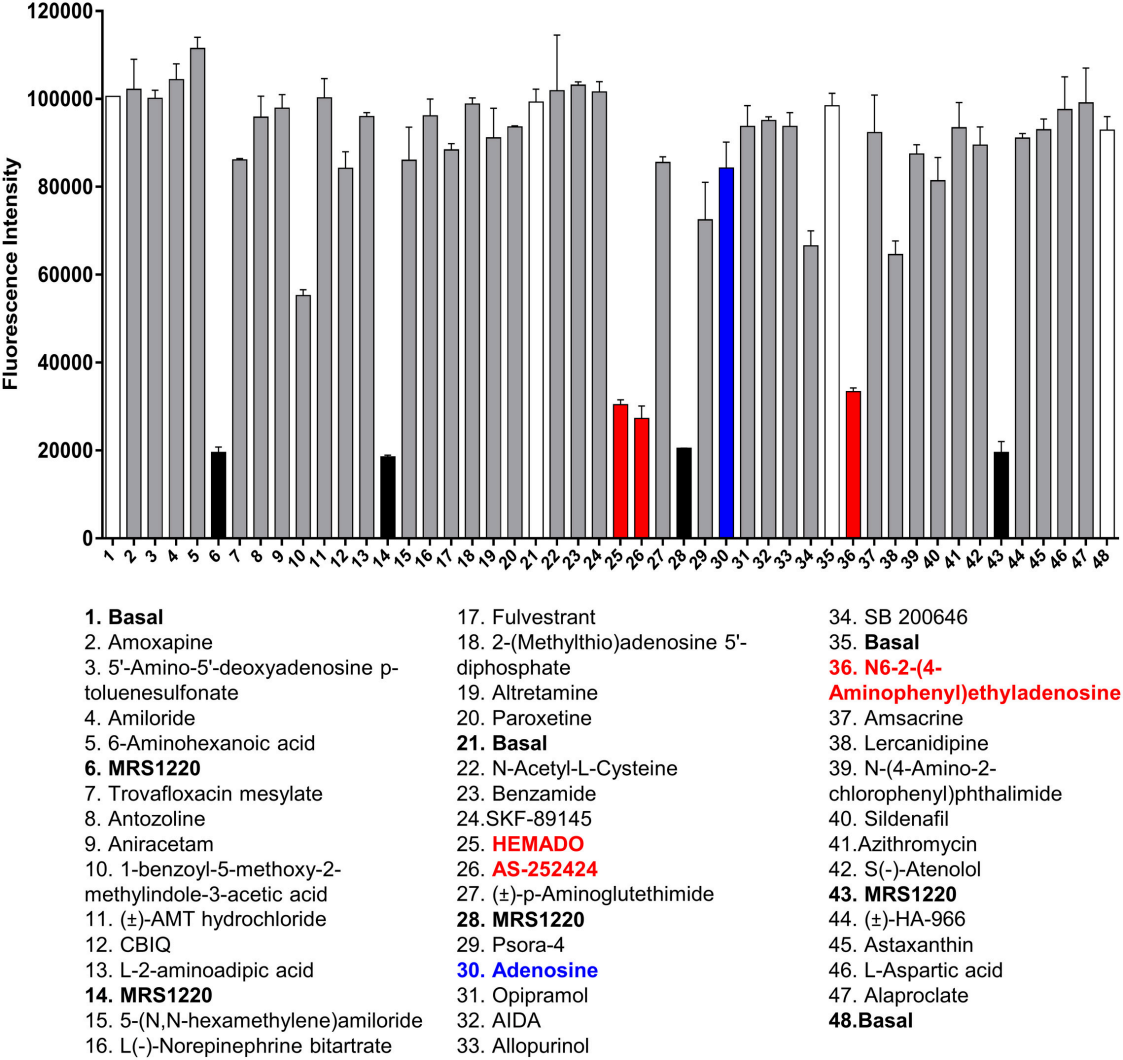
Screening the LOPAC library against the A_3_AR. Example of the data generated from one plate of compounds from the LOPAC library. Each plate contained 40 compounds (each at 10 μM final concentration) from the LOPAC library in duplicate along with four basal and four MRS1220 (10 μM) controls, also in duplicate. The fluorescence intensities obtained on the PHERAstar FS from this plate are shown as mean and range of duplicates with the hits highlighted in red and adenosine indicated in blue. The plate shown is a representative plate of one of the three experiments performed using these compounds and the inhibition data for all compounds screened can be found in Supporting Information Table 1.

To determine whether the HTS version of the competitive fluorescent binding assay was suitable for the screening of large compound libraries, we chose to screen the Library of Pharmacologically Active Compounds (LOPAC) against the A_3_AR. The LOPAC library is considered to be a recognized standard for assay validation as it is based on an extensive number of bioactive compounds. Many of these are known to affect targets involved in adenosine receptor signaling (Iturrioz et al., 2010). CHO cells expressing the A_3_AR were grown to confluency in 96-well plates and incubated with a single concentration (10 μM) of the known A_3_AR antagonist MRS1220 as a positive control or one of the 1,263 compounds (10 μM) from the LOPAC library and CA200645 (25 nM) and the fluorescence intensity of each well determined on the PHERAstar FS plate reader as described in *Experimental Procedures*. Hits were defined as those compounds which inhibited the binding of CA200645 by >40%, and of the initial 1263 compounds evaluated, 67 hits were identified (Supporting Information Table 1, Figure 3, Table 3). Inhibition data for all the compounds tested in the initial screen can be found in Supporting Information Table 1. Among the hits, all the compounds within the library with medium to high affinity for the A_3_AR (pK_i_ ≥6; Figure 3, Table 3) were identified along with four low affinity adenosine-related molecules (1,3-dipropyl-8-*p*-sulfophenylxanthine, DMPX, etazolate hydrochloride and 2-phenylaminoadenosine; Table 3). This confirmed the utility of this approach to identify compounds with known A_3_AR binding affinity. Importantly, the assay Z' factor was 0.47 ± 0.03 (mean ± SEM, *n* = 97), demonstrating its suitability for screening larger libraries in living cells.

**Table 3:** Known A_3_AR ligands in the LOPAC library: Compounds within the LOPAC library that have known activity at adenosine receptors, their rank order in the full screen and the % of 25 nM CA200645 binding in the presence of 10 μM of these compounds.

**Name**	**Agonist or antagonist**	**LOPAC description**	**% Total CA200645 binding**	**Rank**
CGS 15943	Antagonist	Potent non-selective adenosine receptor antagonist	30.0 ± 3.0	9
2-Cl-IB-MECA	Agonist	A_3_ adenosine receptor agonist	32.3 ± 6.1	12
IB-MECA	Agonist	Selective A_3_ adenosine receptor agonist	36.3 ± 4.0	18
NECA	Agonist	Adenosine receptor agonist	38.1 ± 4.3	20
HEMADO	Agonist	A_3_ adenosine receptor agonist	40.1 ± 10.5	24
APNEA	Agonist	Non-selective adenosine receptor agonist	41.0 ± 7.2	26
1,3-dipropyl-8-*p*-sulfophenylxanthine	Antagonist	Adenosine receptor antagonist (slight selectivity for A_1_ over A_2_)	42.3 ± 4.8	29
AB-MECA	Agonist	High affinity A_3_ adenosine receptor agonist	49.5 ± 5.8	38
2-CADO	Agonist	Adenosine receptor agonist with selectivity for A_1_ over A_2_	51.0 ± 6.7	43
SCH 58261	Antagonist	A_2A_ adenosine receptor antagonist	52.2 ± 5.4	47
CV1808	Agonist	Selective A_2_ adenosine receptor agonist	53.3 ± 19.9	56
DPCPX	Antagonist	Selective A1 adenosine receptor antagonist	56.3 ± 3.4	58
FSCPX	Antagonist	Irreversible A_1_ adenosine receptor antagonist	57.5 ± 23.0	63
MRS 1523	Antagonist	Selective A_3_ adenosine receptor antagonist in rat	58.3 ± 11.4	64

**Figure 4:**
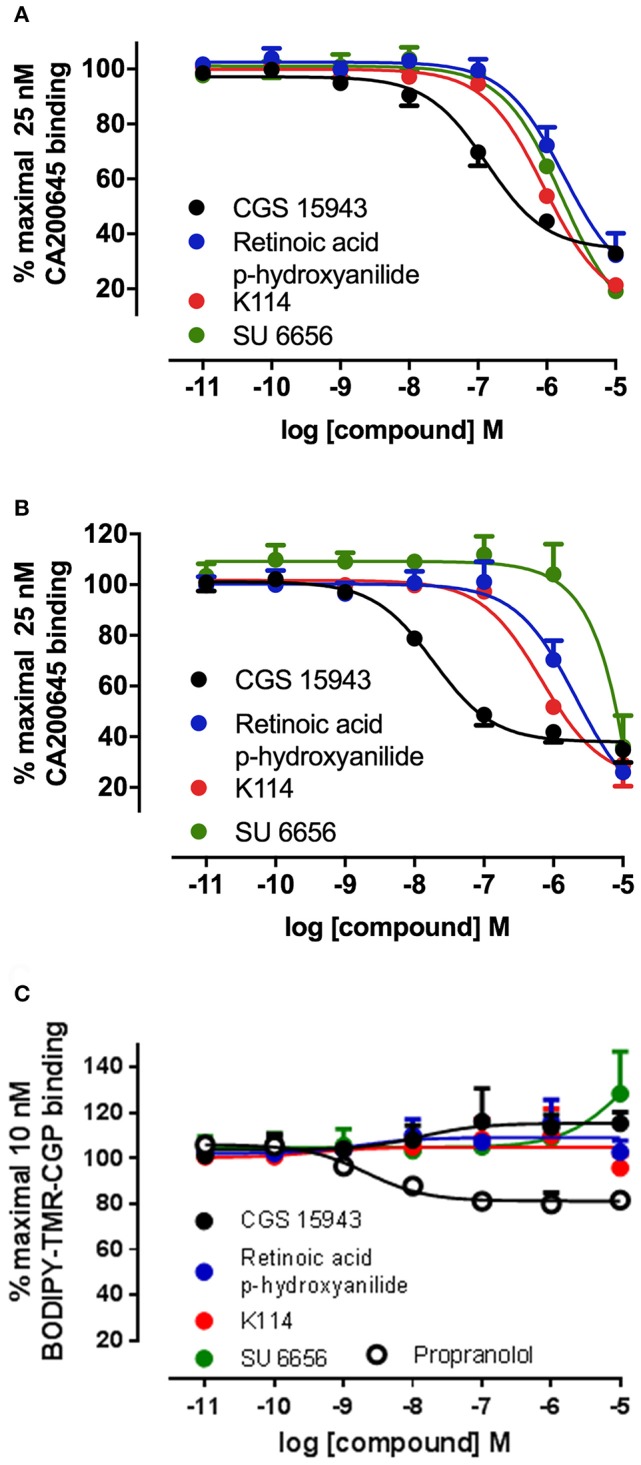
Competition binding curves at the A_1_AR, A_3_AR, and β_2_AR for three hits identified from the LOPAC library. CHO cell lines stably expressing A_1_AR **(A)**, A_3_AR **(B)**, or β_2_AR **(C)** were incubated with 25 nM CA200645 (A_3_AR and A_1_AR) or 10 nM BODIPY-TMR-CGP (β_2_AR) in the absence or in the presence of increasing concentrations of the indicated compounds. Values are mean ± SEM from three independent experiments performed in triplicate.

Ten hits from the initial screen which demonstrated the biggest inhibition of CA200645 binding to the A_3_AR were investigated further and full inhibition curves for each compound were generated. We were unable to further test reactive blue 2 (position 4 in the full screen) as it is currently not available commercially. As shown in Figure 4, Table 4, four of the top ten compounds showed low- to sub-micromolar affinity for the A_3_AR. As expected the adenosine receptor antagonist CGS15943 displaced the binding of CA200645 at both the A_3_AR and A_1_AR in a concentration-dependent manner with the expected affinity (Figure 4, Table 1). As CGS15943 was one of the top ten hits from the initial screen it was also tested in cells expressing the β_2_AR and had no effect on the binding of BODIPY-TMR-CGP (Figure 4). Three further compounds, retinoic acid *p*-hydroxyanilide (fenretinide), K114 and SU 6656, were found to inhibit the binding of CA20065 to the A_3_AR in a concentration-dependent manner with affinity values in the sub-micromolar range, roughly 10-fold lower than CGS15943 (Figure 4, Table 4). Five further hits (BIO, rottlerin, quercetin, PD173952 and kenpaullone) only displaced the binding of CA200645 at the highest concentration tested (10 μM), prohibiting an accurate affinity determination. For those four compounds showing micromolar affinity, the selectivity of their interaction with the A_3_AR was determined by investigating their ability to bind to A_1_AR and β_2_AR. Both K114 and retinoic acid *p*-hydroxyanilide inhibited the binding of CA200645 at the A_1_AR with similar affinity to that observed at the A_3_AR. SU 6656 only inhibited binding at the highest concentration tested and the affinity was not calculated. None of the other compounds showed any measureable activity at the A_1_AR. When tested in CHO cells expressing the β_2_AR, no significant inhibition of BODIPY-TMR-CGP binding was observed for any of the 10 compounds screened but the control β_2_AR antagonist propranolol had the expected affinity (pK_i_ = 8.72 ± 0.14, *n* = 3). There was an increase in fluorescence in the presence of 10 μM SU 6656 (128.4 ± 18.4%). However this was small compared to the increase seen with 10 nM BODIPY-TMR-CGP and the large increase in fluorescence in the presence of BIO (pEC_50_ = 5.84 ± 0.13). This is likely to be due to these compounds interfering with the BODIPY-TMR fluorescence signal, which was not observed when using the more red-shifted BODIPY 630/650 fluorophore in the A_1_AR and A_3_AR binding assays.

**Table 4:** Affinity of selected hits from the LOPAC library at the A_3_AR, A_1_AR, and β_2_AR: Compounds were tested on CHO cells expressing the A_3_AR, A_1_AR, and β_2_AR in the HTS format fluorescent ligand binding assay using 25 nM CA200645 as the tracer for A_3_AR and A_1_AR and 10 nM of BODIPY-TMR-CGP for β_2_AR.

**Position in primary screen**	**Compound**	**A_3_AR**	**A_1_AR**	**β_2_AR**
		**pK_i_**	**pK_i_**	**% Total binding at 10 μM**
2	SU 6656	6.17 ± 0.08	ND	128.4 ± 18.4
5	K114	6.43 ± 0.04	6.56 ± 0.11	95.8 ± 5.5
8	Retinoic acid p-hydroxyanilide	6.13 ± 0.18	6.04 ± 0.21	102.7 ± 5.1
9	CGS 15943	7.24 ± 0.14	8.14 ± 0.09	115.4 ± 5.0

*Data represents mean ± SEM from three experiments performed in triplicate. ND, Not determined as accurate curve could not be generated*.

### Molecular modeling of selected LOPAC hits at the A_3_AR

**Figure 5:**
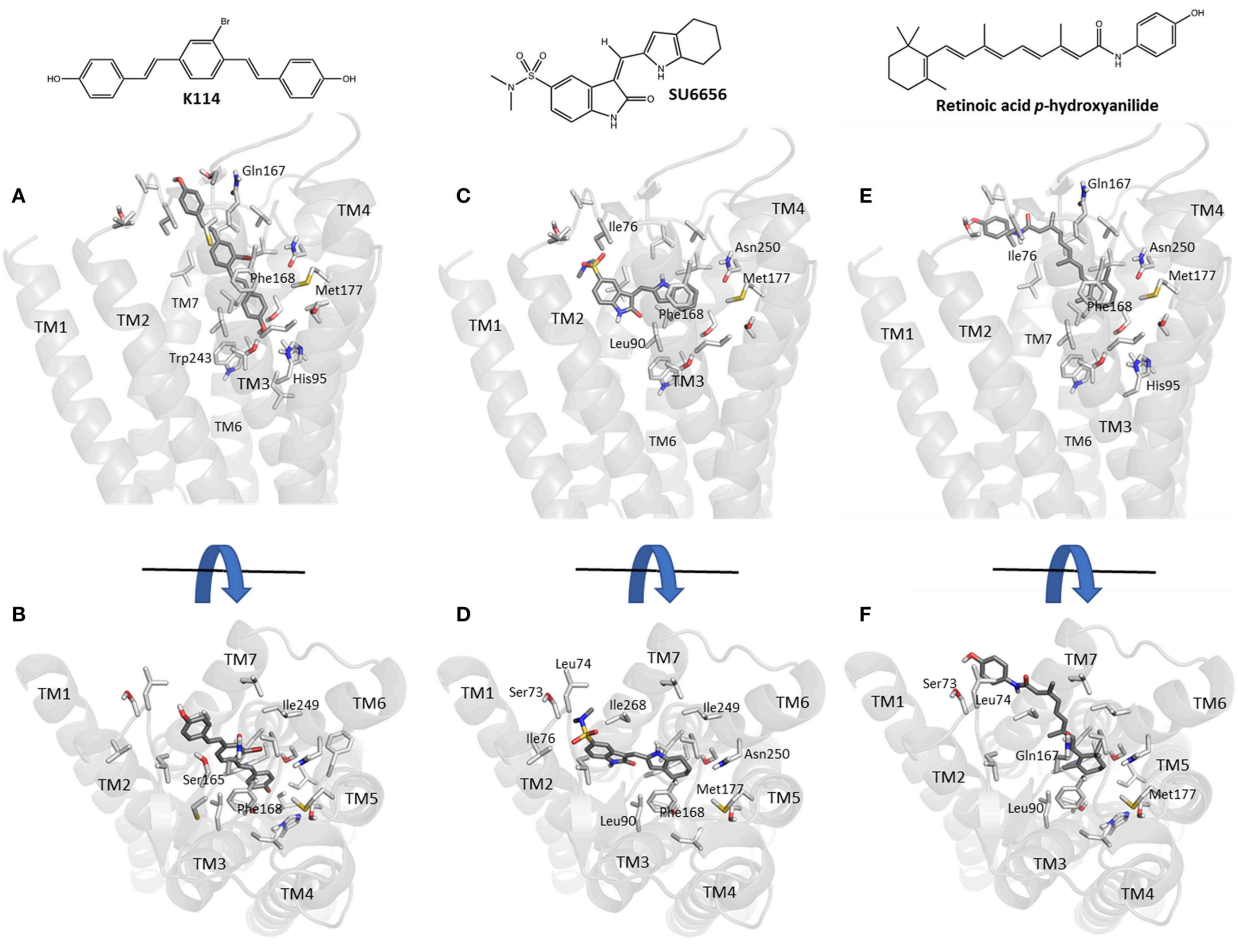
Molecular modeling simulation of K114, SU 6656 and retinoic acid *p*-hydroxyanilide binding to the A_3_AR. A side-on **(A,C,E)** and top-down **(B,D,F)** view of the top scoring binding poses for K114, SU 6656 and retinoic acid *p*-hydroxyanilide (dark gray liquorice coloring) respectively, bound into our previously reported A_3_AR receptor homology model (Vernall et al., 2013). Previously identified amino acid side chain residues associated with the orthosteric binding pocket (Squarcialupi et al., 2013) are represented in light gray liquorice coloring and labeled alongside the TM loop regions for clarity.

Using our previously established homology model of the human A_3_AR (Vernall et al., 2013) we sought to investigate potential binding poses for the three sub-micromolar compounds (retinoic acid *p*-hydroxyanilide (fenretinide), K114 and SU 6656) identified in the LOPAC screen which did not have previous literature precedent for interacting with this receptor sub-type. Using the commercially available docking software, CLC Drug Discovery Workbench, ligand and receptor binding pocket preparation was followed by targeted ligand docking. The highest scoring docked poses for K114, SU 6656 and retinoic acid *p*-hydroxyanilide were selected and are illustrated in Figure 5. All three compounds were able to engage via plausible poses to the A_3_R within the vicinity of the orthosteric binding pocket of this receptor.

## Discussion

Fluorescent ligands for GPCRs are a valuable tool in the study of multiple aspects of receptor pharmacology and they are a potential replacement for radiolabelled ligands in saturation and equilibrium binding studies to determine the affinity of labeled and unlabeled ligands (Stoddart et al., 2016). In this study, we aimed to further develop a previously described fluorescence based live cell binding assay that used a HCS system (Stoddart et al., 2012) to an assay that could be performed with un-tagged receptors on a HTS system. To this end, we chose the PHERAstar FS fluorescent plate reader since it allowed the determination of the optimal focal height for the fluorescence read and multiple scans per well. Use of the HTS system to obtain data resulted in a marked reduction in the time each 96-well plate took to process; from around 40 minutes per plate on the confocal HCS system for data collection and analysis to less than 3 minutes for the HTS system. This also produced a significant reduction in the amount of data that needed to be stored; 500 Mb per plate for HCS versus 160 Kb for HTS. Using the A_3_AR as a model system, we demonstrated that the data generated on the HTS system was in close agreement to that obtained on the HCS system, validating this system as a higher throughput methodology that would be essential for screening large compound libraries using fluorescence-based binding assays in whole cells.

Various methods using fluorescent ligands to measure ligand binding at GPCRs have been recently developed, each using a different approaches to measure the fluorescence of the bound ligand, including flow cytometry (Young et al., 2005; Hara et al., 2009; Kozma et al., 2013), fluorescence polarization (Cornelius et al., 2009; Kecskes et al., 2010) and resonance energy transfer based systems (Zwier et al., 2010; Stoddart et al., 2015a). Each method has advantages and disadvantages, for instance ligand depletion (fluorescence polarization) and the need to tag the receptor of interest (BRET and FRET). One limitation of the simple fluorescent intensity measurement used in the system described here is the potential for a low signal/noise ratio as a result of high levels of non-specific binding and the use of whole cells. As this technique measures total well fluorescence intensity it will be affected by both high levels of non-specific membrane binding and also non-specific uptake of the fluorescent ligand into the cells. As an example of this, for the A_3_AR the maximal reduction in the levels of CA200645 fluorescence measured in the presence of unlabeled ligands was 60% whilst that with BODIPY-TMR-CGP for the β_1_AR was only 20% (Figures 1C, 2B). This small signal/noise ratio for this ligand at the β_1_AR has been observed previously (Gherbi et al., 2014), although it is notable that even under these conditions, the method described here still allowed us to generate robust data within this small signal/noise window. The proximity-based assays (e.g., NanoBRET; Stoddart et al., 2015a) overcome this issue but they obviously require genetic modification of the extracellular N-terminus of the receptor with a fluorescent or luminescent protein, which precludes their use on native receptors—a main aim of the assay developed in this study. What is also clear from this point of view, is that the limit of this signal to noise ratio is likely to be highly dependent on both the pharmacological and photophysical properties of the fluorescent ligand, as we have previously demonstrated (Vernall et al., 2013). To progress the use of this assay to use with endogenously expressed untagged receptors, consideration should also be given to fluorescent ligand selectivity in situations where multiple receptor subtypes are often co-expressed; this is particularly true for adenosine receptors. To this end, the demonstration that this assay also works with a highly A_3_AR selective ligand, AV039 (Vernall et al., 2012) is important.

To demonstrate the utility of this assay system for compound screening, we investigated if we could identify known ligands for the A_3_AR within a library of pharmacologically active compounds (LOPAC). Within the LOPAC library there were 37 compounds identified as ligands for adenosine receptors. For the 1,263 compounds screened, we defined a hit as a compound that inhibited more than 40% of the total CA200645 binding. Using these criteria, we identified 67 hits, of which 14 had previously described activity at adenosine receptors (Table 3). Of these, four were the known A_3_R selective agonists, 2-Cl-IB-MECA (Gallo-Rodriguez et al., 1994), IB-MECA (Klotz et al., 1998), AB-MECA (Klotz et al., 1998) and HEMADO (Klotz et al., 2007), and the A_3_R selective antagonist MRS1523 (Li et al., 1998). A further five compounds were known to be non-selective at this adenosine receptor subtype [CGS15943 (Ongini et al., 1999), NECA (Gao et al., 2004), APNEA (Gao et al., 2004), 2-CADO (van Galen et al., 1994) and 1,3-dipropyl-8-*p*-sulfophenylxanthine (Daly et al., 1985)]. The remaining four compounds were SCH 58261, CV1808, DPCPX and FSCPX. SCH 58261 is widely described as an A_2A_ selective and DPCPX as an A_1_AR-selective antagonist, and both retain affinity in the μM range for the A_3_AR (Ongini et al., 1999; Stoddart et al., 2012). FSCPX is an irreversible antagonist at the A_1_AR (van Muijlwijk-Koezen et al., 2001) but to date it had not been tested at other adenosine receptor subtypes. Our data from this screen indicates that FSCPX is likely to retain activity at the A_3_R at least in the low μM range and this is also true for CV1808 that has been described as an agonist at the A_2A_AR (Dionisotti et al., 1997). A variety of different compounds that act at different (i.e., non-A_3_AR) adenosine receptors were included in the library and as expected were not identified as hits in our screen (Supporting Information Table 1). These included A_1_AR selective agonists and antagonists such as R-PIA (Klotz et al., 1998) and CPT (Dalpiaz et al., 1998), A_2A_AR selective agonists and antagonists such as CGS 21680 (Klotz et al., 1998) and CSC (Jacobson et al., 1993), and the A_2B_AR selective antagonist alloxazine (Ji et al., 2001). A variety of low affinity non-selective antagonists and agonists were also present in the library including adenosine, theophylline, caffeine and paraxanthine that have reported affinity at the A_3_AR in the 13-100 μM range (Jacobson et al., 1999; Fredholm et al., 2001). Due to the concentration of CA200645 (25 nM) used in the primary screen only compounds with an affinity of <10 μM would be expected to be identified as a hit. Overall, the assay performed well at identifying all the compounds with known activity at the A_3_AR.

We found three compounds in the library that displayed unexpected sub-micromolar affinity at the A_3_AR (Figure 4, Table 4). These were K114, retinoic acid *p*-hydroxyanilide and SU 6556. K114 is used to identify amyloid lesions from Aβ peptide, α-synuclien and tau through an increase in its fluorescence upon binding to these lesions. It is has minimal fluorescence in aqueous solution and has emission maxima of 550 nm that is unlikely to interfere with the emission of BY630 at 650 nm (Crystal et al., 2003). In addition, the assay described here monitors a decrease in fluorescence in the presence of inhibitors that would mean it would be more likely to give false-negatives rather than false-positives. Retinoic acid *p*-hydroxyanilide, also known as fenretinide or 4-HPR, is an analog of retinoic acid and is a potential therapy in the treatment of cancer due to its ability to induce apoptosis (Wu et al., 2001). It is possible that it was causing apoptosis of the cells in our assay system leading to a concurrent decrease in fluorescence but as the presence of retinoic acid *p*-hydroxyanilide had no effect in cells expressing the β_2_AR this is unlikely to be the case (Figure 4). SU6556 is a Src kinase inhibitor that has also been found to inhibit a variety of other kinases including Aurora C and AMPK (Bain et al., 2007). It also displayed slight selectivity for the A_3_AR over A_1_AR.

Docking of the sub-micromolar compounds identified in the LOPAC screen provided a plausible set of binding poses within the vicinity of the established orthosteric A_3_AR binding pocket (Figure 5). K114 bound in a fully extended form with one of the terminal phenols optimally positioned to engage in a hydrogen bond interaction with the side-chain of Thr94. Meanwhile, the remaining vinyl-linked aromatic moieties pass through a hydrophobic channel created by Ile76, Val169, Leu90, Leu246, Ile249, Leu264, Ile268, and Phe168; the latter engaging via a face-to-face pi-stacking interaction. SU 6656 favored binding higher up in the orthosteric pocket with the 4,5,6,7-tetrahydroindolyl portion of the molecule engaging in a face-to-face interaction with Phe168, with the hydrophobic interactions predominating with Leu90, Val65, Ile268, and Leu246. Finally, retinoic acid *p-*hydroxyanilide displayed a binding pose passing through the same hydrophobic channel observed with K114. The 1,3,3-trimethylcyclohex-1-enyl region of the molecule was positioned deepest into the binding pocket engaging in hydrophobic interactions with residues Leu246, Ile249, Met177, and Phe168. The *p*-hydroxyanilde region of the molecule was positioned in such a way as to allow a face-to-edge interaction with Tyr265 at the top of transmembrane helix 7. With the predominance of aromatic and hydrophobic interactions observed between the receptor and the three ligands discussed, this would seem to correlate well with the experimental binding affinities whilst also offering the potential to undertake productive modifications of these compounds to potentially enhance their overall binding interactions.

In conclusion, we have shown that a simple intensity based fluorescent ligand binding assay can be modified to work in a potentially high throughput format, giving significant advances in both speed and data volume compared to previous high content versions. The assay allows screening of a small compound library in live cells, and can assess binding to the unmodified native receptors. The assays performed well under test conditions, identifying both known adenosine receptor ligands in a focused library as well as novel potential ligand scaffolds. Further work on establishing this assay to screen at endogenous A_3_AR in a mixed receptor background will be important to allow subsequent screens to be performed under more physiological conditions.

## Experimental procedures

### Chemicals

Known GPCR antagonists were purchased from Tocris Bioscience and G418 was obtained from Invitrogen. Fetal calf serum was obtained from PAA Laboratories and L-glutamine from Lonza. All other biological reagents were obtained from Sigma-Aldrich. CA200645 was obtained from CellAura Technologies. BODIPY-TMR-CGP (BODIPY-TMR-(±)-CGP 12177) was purchased from Molecular Probes. AV039 and AV019 were synthesized in house as previously described (Vernall et al., 2012). The LOPAC library was obtained from Sigma-Aldrich.

### Cell culture

CHO-K1 cells stably expressing the human A_3_AR (Vernall et al., 2012), β_1_AR (Guo et al., 2012), β_2_AR (Baker et al., 2002) or the human A_1_AR (May et al., 2010) were maintained in DMEM/F12 medium containing 10% fetal calf serum and 2 mM L-glutamine at 37°C in a humidified atmosphere of air/CO_2_ (19:1).

### Fluorescence competition binding assay

CHO cells stably expressing the A_3_AR, A_1_AR, β_1_AR or β_2_AR were seeded into the central 60 wells (for high content confocal analysis) or every well (high throughput analysis) of a 96-well clear-bottomed, black-walled plate (Greiner BioOne) and grown to confluency. On the day of experiment, normal growth medium was removed and cells washed twice with HEPES-buffered saline solution (HBSS; 10 mM HEPES, 10 mM glucose, 145 mM NaCl, 5 mM KCl, 1 mM MgSO_4_, 2 mM sodium pyruvate, 1.3 mM CaCl_2_, 1.5 mM NaHCO_3_, pH 7.4) pre-warmed to 37°C. Fresh HBSS was added to each well followed by the addition of the required concentration of unlabeled compound and the respective fluorescent ligands (25 nM CA200645, 5 nM AV039 or 10 nM BODIPY-TMR-CGP). Cells were incubated for 1h at 37°C/5% CO_2_. Buffer was then removed from each well, cells washed once in HBSS and fresh HBSS added at room temperature. Plates were then immediately subjected to high content or HTS analysis as detailed below.

### High content screening

High content analysis was conducted as previously described (Stoddart et al., 2012). Briefly, plates were imaged using an ImageXpress Ultra confocal plate reader, which captured four central images per well using a Plan Fluor 40x NA0.6 extra-long working distance objective. CA200645 was excited at 635 nm and emission collected through a 640–685 nm band pass filter. Total image intensity was obtained using a modified multi-wavelength cell scoring algorithm within the MetaXpress software (MetaXpress 2.0, Molecular Devices).

### High throughput screening

High throughput analysis was performed using a PHERAstar FS plate reader (BMG Technologies). Fluorescent intensity of each well was assessed by bottom scanning using the following optical modules: excitation 540 nm and emission 590 nm (for BODIPY-TMR-CGP-labeled cells), or excitation 630 nm and emission 650 nm (for the BY630 compounds CA200645 and AV039). Optimal focal height was determined automatically and total fluorescence intensity was assessed by taking 81 reads per well.

### Screening of the LOPAC library of pharmacological active compounds

The LOPAC compound library contained 1263 compounds and each compound was provided as a pre-dissolved solution in 10 mM in DMSO. Compound plates containing 2 μl of compound per well were provided by the University of Nottingham Managed Compound Collection. Each plate contained 40 compounds from the LOPAC library together with positive and blank control samples. For the blank controls, 2 μl of DMSO was added per well and for the positive controls the A_3_AR antagonist MRS1220 (10 μM final concentration) was used. The compounds were diluted to 100 μM in HBSS prior to assay. Each compound was tested in duplicate at a final concentration of 10 μM on three separate experimental days. Experiment was carried out as detailed above using the A_3_AR expressing cell line and 25 nM CA200645 as the tracer ligand. Data were normalized on a per plate basis to the fluorescence observed in blank control wells.

The 67 compounds that inhibited by more than 40% the total binding of CA200645 compared to blank controls were classed as hits. From this list 16 compounds were selected for secondary screening to determine their IC_50_ values and binding affinity. This was achieved by investigating the effect of increasing concentrations of each inhibitor on the specific binding of 25 nM CA200645 or 10 nM BODIPY-TMR-CGP in cells expressing the A_3_AR, A_1_AR or β_2_AR.

### Molecular modeling

Using our previously reported homology model of the human A_3_AR (Vernall et al., 2013) and the CLC Drug Discovery Workbench software package (Version 3.0.2, Qiagen, Netherlands), the protein target was prepared with no water molecules present. Before setting up the docking experiments, the binding site was generated as a 13 Å sphere centered around the established orthosteric pocket. All small molecules were constructed using ChemDraw Professional 16.0 (CambridgeSoft, Cambridge, MA, USA) and imported into the docking programme using the Balloon PlugIn (http://users.abo.fi/mivainio/balloon) (Vainio and Johnson, 2007) to afford the lowest energy conformer for each ligand. During the docking process, each ligand underwent 1000 individual iterations, with the conformation of each ligand set as flexible, allowing full movement around all rotatable bonds, whilst the protein was held as a rigid structure. The best scoring pose for each ligand was returned using the PLANTS_PLP_ algorithm to determine that docking score (Korb et al., 2009) and the best ranked compounds were selected and their binding residues observed using the CLC Drug Discovery Workbench visualization tool.

### Data analysis

Competition binding curves were fitted to the following equation using GraphPad Prism 5 (GraphPad Software):

$$\begin{array}{l} \%\;inhibition\;of\;specific\;binding=\frac{100\times \left[A\right]}{\left[A\right]+IC_{50}} \end{array}$$

where [A] is the concentration of competing drug and IC_50_ is the molar concentration of ligand required to inhibit 50% of the specific binding of a fixed concentration [L] of the appropriate fluorescent ligand. The IC_50_ values obtained were converted to K_i_ values using the following equation:

$$\begin{array}{l} K_{i}=\;\frac{IC_{50}}{1+\;\frac{\left[L\right]}{K_{D}}} \end{array}$$

where [L] is the concentration and K_D_ is the equilibrium dissociation constant of the fluorescent ligand. The K_D_ values for the fluorescent ligands used were 11.0 nM and 3.11 nM for CA200645 at the A_1_AR and A_3_AR respectively (Stoddart et al., 2012). K_D_ values for BODIPY-TMR-CGP were taken from Baker et al. (2003).

The Z′ values were calculated on a per plate basis using the following equation:

$$\begin{array}{l} Z^{\prime }=1-\;\frac{3\left(\sigma_{p}+\;\sigma_{n}\right)}{\mu_{p\;}-\;\mu_{n}} \end{array}$$

where μ_p_ and σ_p_ are the mean and standard deviation from the control wells (DMSO only) and μ_n_ and σ_n_ are the mean and standard deviation from the MRS1220 treated wells.

## Author contributions

SH, SB, and BK: Conceived the study; MA, LS, SB, BK, and SH: Participated in research design; MA and LS: Performed the experiments and data analysis; KG: Performed the beta receptor screening experiments and analyzed the data; BK: Performed the molecular docking studies; MA, LS, BK, SB, and SH: All wrote or contributed to the writing and editing of the manuscript.

### Conflict of interest statement

The authors declare that the research was conducted in the absence of any commercial or financial relationships that could be construed as a potential conflict of interest.
