# Supplementary material for: A Non-imaging High Throughput Approach to Chemical Library Screening at the Unmodified Adenosine-A3 Receptor in Living Cells
Source: Front Pharmacol. 2017 Dec 13;8:908. doi: 10.3389/fphar.2017.00908 (PMC5733478; doi:10.3389/fphar.2017.00908)
Supplement: Supplementary file 1 [file Table1.DOCX]

**Supporting Information**

**for**

**A non-imaging high throughput approach to chemical library screening at the unmodified adenosine-A_3_ receptor in living cells**

Maria Augusta Arruda^*^, Leigh A Stoddart^*^, Karolina Gherbi, Stephen J Briddon, Barrie Kellam, Stephen J Hill

**Table 1. Inhibition of CA200645 binding at the A_3_AR by the LOPAC library of compounds.**

Values obtained in a fluorescent adenosine receptor antagonist binding assay using whole, live cells expressing the A_3_AR. Values quoted are % of control wells (wells containing 1% DMSO and 25 nM CA200645). All compounds were tested at 10 µM. Data shown represents mean ± SD from three separate experiments performed in duplicate. ND = not determined as compounds were not included in the screen.

| **Rank** | **Compound Name** | **% 25 nM CA200645 binding** |  | **Rank** | **Compound Name** | **% 25 nM CA200645 binding** |
| --- | --- | --- | --- | --- | --- | --- |
| 1 | BIO | 16.1 ± 3.2 |  | 641 | 1-(4-Chlorobenzyl)-5-methoxy-2-methylindole-3-acetic acid | 97.1 ± 10.6 |
| 2 | SU 6656 | 17.3 ± 1.8 |  | 642 | Etodolac | 97.1 ± 13.7 |
| 3 | Rottlerin | 22.8 ± 1.4 |  | 643 | Anisotropine methyl bromide | 97.1 ± 12.3 |
| 4 | Reactive Blue 2 | 24.5 ± 2.7 |  | 644 | Metrazoline oxalate | 97.1 ± 2.0 |
| 5 | K114 | 24.6 ± 5.8 |  | 645 | Ebastine | 97.2 ± 6.4 |
| 6 | Quercetin dihydrate | 25.2 ± 6.9 |  | 646 | (+)-Brompheniramine maleate | 97.2 ± 7.4 |
| 7 | PD173952 | 25.6 ± 5.3 |  | 647 | Citalopram hydrobromide | 97.2 ± 4.4 |
| 8 | Retinoic acid p-hydroxyanilide | 26.5 ± 2.0 |  | 648 | 1,5-Isoquinolinediol | 97.2 ± 4.1 |
| 9 | CGS-15943 | 30.0 ± 3.0 |  | 649 | Paroxetine hydrochloride hemihydrate | 97.2 ± 4.3 |
| 10 | Kenpaullone | 30.6 ± 6.6 |  | 650 | S(-)-Atenolol | 97.2 ± 11.6 |
| 11 | DAPH | 31.8 ± 9.9 |  | 651 | (±)-CPP | 97.2 ± 1.5 |
| 12 | Chloro-IB-MECA | 32.3 ± 6.1 |  | 652 | Captopril | 97.2 ± 6.3 |
| 13 | PD-166866 | 34.3 ± 9.7 |  | 653 | U0126 | 97.2 ± 19.0 |
| 14 | Rutaecarpine | 34.3 ± 2.1 |  | 654 | 8-(p-Sulfophenyl)theophylline | 97.2 ± 13.7 |
| 15 | PD 169316 | 35.7 ± 3.3 |  | 655 | Nisoxetine hydrochloride | 97.3 ± 6.5 |
| 16 | 1,3,5-tris(4-hydroxyphenyl)-4-propyl-1H-pyrazole | 35.7 ± 9.6 |  | 656 | Imiloxan hydrochloride | 97.3 ± 10.2 |
| 17 | AGK2 | 35.8 ± 5.6 |  | 657 | CHM-1 hydrate | 97.3 ± 5.3 |
| 18 | IB-MECA | 36.3 ± 4.0 |  | 658 | IMID-4F hydrochloride | 97.3 ± 5.7 |
| 19 | U-74389G maleate | 36.9 ± 10.8 |  | 659 | SKF-89145 hydrobromide | 97.3 ± 9.6 |
| 20 | 5'-N-Ethylcarboxamidoadenosine | 38.1 ± 4.3 |  | 660 | (±)-Methoxyverapamil hydrochloride | 97.3 ± 10.6 |
| 21 | CL 316,243 | 38.7 ± 18.1 |  | 661 | Venlafaxine hydrochloride | 97.3 ± 8.5 |
| 22 | Calcimycin | 38.9 ± 3.3 |  | 662 | CGS-12066A maleate | 97.3 ± 10.3 |
| 23 | Sanguinarine chloride | 38.9 ± 20.6 |  | 663 | Vinpocetine | 97.3 ± 11.5 |
| 24 | HEMADO | 40.1 ± 10.4 |  | 664 | Sunitinib malate | 97.4 ± 12.4 |
| 25 | SP600125 | 41.0 ± 10.4 |  | 665 | Imazodan | 97.4 ± 12.7 |
| 26 | N6-2-(4-Aminophenyl)ethyladenosine | 41.0 ± 7.1 |  | 666 | Atropine sulfate | 97.4 ± 1.9 |
| 27 | 6(5H)-Phenanthridinone | 41.6 ± 14.1 |  | 667 | DL-Cycloserine | 97.4 ± 8.2 |
| 28 | Apigenin | 41.8 ± 13.4 |  | 668 | (±)-Vanillylmandelic acid | 97.4 ± 12.9 |
| 29 | 1,3-Dipropyl-8-p-sulfophenylxanthine | 42.3 ± 4.8 |  | 669 | Sepiapterin | 97.4 ± 20.7 |
| 30 | SU 5416 | 43.3 ± 10.8 |  | 670 | Albuterol hemisulfate | 97.4 ± 12.0 |
| 31 | DL-Stearoylcarnitine chloride | 44.2 ± 6.0 |  | 671 | 4-Aminobenzamidine dihydrochloride | 97.4 ± 8.5 |
| 32 | Roscovitine | 45.3 ± 8.6 |  | 672 | Diltiazem hydrochloride | 97.4 ± 8.9 |
| 33 | AS-252424 | 45.8 ± 20.9 |  | 673 | CGP-13501 | 97.4 ± 7.1 |
| 34 | Etazolate hydrochloride | 46.2 ± 5.3 |  | 674 | L-741,626 | 97.5 ± 15.5 |
| 35 | Eupatorin | 47.2 ± 12.7 |  | 675 | Sematilide monohydrochloride monohydrate | 97.5 ± 2.4 |
| 36 | Imperatorin | 47.6 ± 2.3 |  | 676 | Tomoxetine | 97.5 ± 8.3 |
| 37 | AB-MECA | 48.5 ± 9.1 |  | 677 | 1-Allyl-3,7-dimethyl-8-p-sulfophenylxanthine | 97.5 ± 9.3 |
| 38 | Furafylline | 49.5± 5.8 |  | 678 | Gabaculine hydrochloride | 97.5 ± 8.4 |
| 39 | SB 242084 dihydrochloride hydrate | 49.6± 12.4 |  | 679 | Eprosartan mesylate | 97.5 ± 15.0 |
| 40 | MNS | 50.1 ± 14.8 |  | 680 | Labetalol hydrochloride | 97.5 ± 13.9 |
| 41 | Indirubin-3'-oxime | 50.6 ± 24.7 |  | 681 | Cantharidic Acid | 97.5 ± 13.0 |
| 42 | PD-184161 | 50.8 ± 14.3 |  | 682 | SCH-28080 | 97.5 ± 14.8 |
| 43 | 2-Chloroadenosine | 51.0 ± 6.7 |  | 683 | Bendamustine hydrochloride | 97.6 ± 4.1 |
| 44 | SB 218795 | 51.1 ± 8.4 |  | 684 | Chlorpropamide | 97.6 ± 7.8 |
| 45 | Diacylglycerol Kinase Inhibitor II | 51.5 ± 9.4 |  | 685 | Oxaprozin | 97.6 ± 6.3 |
| 46 | (±)-2-Amino-7-phosphonoheptanoic acid | 52.2 ± 10.8 |  | 686 | Agmatine sulfate | 97.6 ± 11.6 |
| 47 | UCL 2077 | 52.2 ± 5.4 |  | 687 | PMEG hydrate | 97.6 ± 14.8 |
| 48 | SCH 58261 | 52.5 ± 9.7 |  | 688 | gamma-Acetylinic GABA | 97.6 ± 3.0 |
| 49 | Emodin | 52.8± 4.4 |  | 689 | Carboplatin | 97.7 ± 5.9 |
| 50 | SU 4312 | 53.2 ± 16.7 |  | 690 | DBO-83 | 97.7 ± 11.0 |
| 51 | N-Oleoyldopamine | 53.5 ± 8.3 |  | 691 | L(-)-Norepinephrine bitartrate | 97.7 ± 6.2 |
| 52 | NU2058 | 53.9 ± 7.1 |  | 692 | loxoprofen | 97.7. ± 0.7 |
| 53 | Gossypol | 54.1 ± 12.3 |  | 693 | Podophyllotoxin | 97.7 ± 17.3 |
| 54 | Calmidazolium chloride | 54.4 ± 19.1 |  | 694 | 5-Hydroxy-L-tryptophan | 97.7 ± 1.5 |
| 55 | PF-573228 | 54.7 ± 30.2 |  | 695 | Atorvastatin calcium salt trihydrate | 97.7 ± 2.2 |
| 56 | 2-Phenylaminoadenosine | 55.3 ± 19.9 |  | 696 | Moclobemide | 97.8 ± 6.0 |
| 57 | GW7647 | 55.8 ± 12.2 |  | 697 | Piribedil maleate | 97.8 ± 1.7 |
| 58 | 8-Cyclopentyl-1,3-dipropylxanthine | 56.3 ± 3.4 |  | 698 | (-)-Naproxen sodium | 97.8 ± 4.0 |
| 59 | Nifedipine | 56.6 ± 11.1 |  | 699 | 5-Aminovaleric acid hydrochloride | 97.9 ± 5.1 |
| 60 | FSCPX | 57.1 ± 9.1 |  | 700 | SKF 83959 hydrobromide | 97.9 ± 6.2 |
| 61 | MRS 1523 | 57.3 ± 10.9 |  | 701 | N-Bromoacetamide | 97.9 ± 5.0 |
| 62 | GW2974 | 57.3 ± 11.9 |  | 702 | BIX 01294 trihydrochloride hydrate | 97.9 ± 21.2 |
| 63 | Tyrphostin AG 879 | 57.5 ± 23.0 |  | 703 | Oxiracetam | 97.9 ± 12.4 |
| 64 | AS 604850 | 58.3 ± 11.4 |  | 704 | S(-)-Pindolol | 98.0 ± 8.4 |
| 65 | 7-Cyclopentyl-5-(4-phenoxy)phenyl-7H-pyrrolo[2,3-d]pyrimidin-4-ylamine | 58.8 ± 13.2 |  | 705 | Amisulpride | 98.0 ± 5.3 |
| 66 | 1-benzoyl-5-methoxy-2-methylindole-3-acetic acid | 59.0 ± 9.2 |  | 706 | L-Cycloserine | 98.0 ± 1.4 |
| 67 | AMG 9810 | 59.0 ± 6.4 |  | 707 | (±)-7-Hydroxy-DPAT hydrobromide | 98.0 ± 1.6 |
| 68 | (+)-Bromocriptine methanesulfonate | 60.6 ± 9.2 |  | 708 | 3-Isobutyl-1-methylxanthine | 98.0 ± 7.1 |
| 69 | SB 206553 hydrochloride | 60.7 ± 9.3 |  | 709 | SB-215505 | 98.1 ± 14.8 |
| 70 | N6-Methyladenosine | 61.1 ± 11.7 |  | 710 | Fluphenazine dihydrochloride | 98.1 ± 12.0 |
| 71 | IRAK-1/4 Inhibitor I | 61.3 ± 9.4 |  | 711 | Demeclocycline hydrochloride | 98.1 ± 10.5 |
| 72 | TNP | 61.8 ± 11.7 |  | 712 | L-Buthionine-sulfoximine | 98.1 ± 5.3 |
| 73 | Myricetin | 62.7 ± 9.0 |  | 713 | cis(+/-)-8-OH-PBZI hydrobromide | 98.1 ± 5.1 |
| 74 | IPA-3 | 63.1 ± 15.2 |  | 714 | Cytosine-1-beta-D-arabinofuranoside hydrochloride | 98.1 ± 3.7 |
| 75 | LY-367,265 | 64.8 ± 25.8 |  | 715 | EBPC | 98.2 ± 13.5 |
| 76 | O6-benzylguanine | 65.0 ± 6.0 |  | 716 | Quinacrine dihydrochloride | 98.2 ± 4.9 |
| 77 | Thapsigargin | 65.1 ± 10.7 |  | 717 | Vinblastine sulfate salt | 98.2 ± 16.0 |
| 78 | YC-1 | 65.3 ± 4.7 |  | 718 | N-Oleoylethanolamine | 98.2 ± 10.8 |
| 79 | Mecamylamine hydrochloride | 65.4 ± 10.0 |  | 719 | Guanabenz acetate | 98.2 ± 11.7 |
| 80 | CGS-21680 hydrochloride | 65.7 ± 8.4 |  | 720 | Tetrahydrozoline hydrochloride | 98.2 ± 6.4 |
| 81 | Genistein | 66.1 ± 12.2 |  | 721 | BRL 37344 sodium | 98.2 ± 9.4 |
| 82 | Psora-4 | 66.4 ± 9.2 |  | 722 | CP-346086 dihydrate | 98.2 ± 12.5 |
| 83 | Mephetyl tetrazole | 66.4 ± 18.5 |  | 723 | (±)-8-Hydroxy-DPAT hydrobromide | 98.2 ± 1.8 |
| 84 | G15 | 66.5 ± 16.0 |  | 724 | Tyrphostin AG 537 | 98.3 ± 17.8 |
| 85 | Fusaric acid | 66.5 ± 29.0 |  | 725 | BU99006 | 98.3 ± 5.1 |
| 86 | Cilnidipine | 67.0 ± 19.0 |  | 726 | Actinonin | 98.3 ± 4.3 |
| 87 | WIN 62,577 | 67.3 ± 5.6 |  | 727 | HA-100 | 98.3 ± 9.4 |
| 88 | (-)-Bicuculline methbromide, 1(S), 9(R) | 67.4 ± 5.9 |  | 728 | Ammonium pyrrolidinedithiocarbamate | 98.3 ± 7.2 |
| 89 | TBB | 67.4 ± 13.4 |  | 729 | Famotidine | 98.3 ± 15.4 |
| 90 | Phloretin | 67.7 ± 15.2 |  | 730 | Pancuronium bromide | 98.3 ± 10.5 |
| 91 | 7,8-Dihydroxyflavone hydrate | 68.2 ± 13.0 |  | 731 | 1,10-Diaminodecane | 98.3 ± 12.0 |
| 92 | CCT007093 | 68.4 ± 3.4 |  | 732 | Sodium Taurocholate hydrate | 98.3 ± 7.3 |
| 93 | SB 202190 | 68.5 ± 12.1 |  | 733 | Bestatin hydrochloride | 98.3 ± 9.7 |
| 94 | S(-)-p-Bromotetramisole oxalate | 68.6 ± 44.8 |  | 734 | Clodronic acid | 98.4 ± 3.5 |
| 95 | CyPPA | 68.8 ± 15.9 |  | 735 | Betaxolol hydrochloride | 98.4 ± 4.9 |
| 96 | Cisplatin | 69.0 ± 8.2 |  | 736 | N-Desmethylclozapine | 98.4 ± 14.7 |
| 97 | R(-)-N6-(2-Phenylisopropyl)adenosine | 69.2 ± 26.4 |  | 737 | D-ribofuranosylbenzimidazole | 98.4 ± 15.0 |
| 98 | N6-Cyclopentyladenosine | 69.6 ± 6.1 |  | 738 | ATPO | 98.4 ± 3.3 |
| 99 | AA-861 | 69.6 ± 8.4 |  | 739 | RepSox | 98.5 ± 5.4 |
| 100 | 6-Hydroxy-DL-DOPA | 69.7 ± 9.9 |  | 740 | Parthenolide | 98.5 ± 15.3 |
| 101 | KRM-III | 70.4 ± 12.2 |  | 741 | SIB 1757 | 98.5 ± 4.5 |
| 102 | R(-)-Apocodeine hydrochloride | 70.5 ± 34.0 |  | 742 | DL-erythro-Dihydrosphingosine | 98.5 ± 11.2 |
| 103 | I-OMe-Tyrphostin AG 538 | 71.3 ± 34.4 |  | 743 | Thiolactomycin | 98.5 ± 7.2 |
| 104 | 1-(1-Naphthyl)piperazine hydrochloride | 71.5 10.7 |  | 744 | p-Fluoro-L-phenylalanine | 98.5 ± 8.4 |
| 105 | PD-156707 | 71.9 ± 8.4 |  | 745 | LE 300 | 98.5 ± 4.5 |
| 106 | Morin | 72.1 ± 9.6 |  | 746 | 1-Deoxynojirimycin hydrochloride | 98.5 ± 11.6 |
| 107 | Ro 90-7501 | 72.1 ± 6.0 |  | 747 | Disopyramide phosphate | 98.5 ± 7.6 |
| 108 | (±)-Chloro-APB hydrobromide | 72.6 ± 20.5 |  | 748 | (-)-Scopolamine,n-Butyl-, bromide | 98.5 ± 15.4 |
| 109 | Celecoxib | 72.6 ± 20.4 |  | 749 | CP-100263 dihydrochloride hydrate | 98.5 ± 6.9 |
| 110 | Indomethacin | 72.9 ± 18.3 |  | 750 | L-allylglycine | 98.5 ± 1.7 |
| 111 | U-73122 | 73.1 ± 7.4 |  | 751 | Nomifensine maleate | 98.5 ± 14.7 |
| 112 | Tyrphostin AG 835 | 73.1 ± 6.8 |  | 752 | Succinylcholine chloride | 98.6 ± 15.7 |
| 113 | Chelerythrine chloride | 73.9 ± 3.7 |  | 753 | EGTA | 95.6 ± 5.9 |
| 114 | Clotrimazole | 74.0 ± 15.0 |  | 754 | 4-Imidazoleacrylic acid | 95.6 ± 8.4 |
| 115 | FPL 64176 | 74.2 ± 6.7 |  | 755 | Cetirizine dihydrochloride | 98.6 ± 19.3 |
| 116 | TBBz | 74.5 ± 15.0 |  | 756 | (+)-Butaclamol hydrochloride | 98.6 ± 1.9 |
| 117 | AL-8810 | 75.0 ± 17.3 |  | 757 | (-)-Isoproterenol hydrochloride | 98.6 ± 14.4 |
| 118 | Flupirtine maleate | 75.3 ± 9.1 |  | 758 | Y-27632 dihydrochloride | 98.6 ± 10.3 |
| 119 | Dephostatin | 75.4 ± 19.6 |  | 759 | Zonisamide sodium | 98.6 ± 10.8 |
| 120 | Cilostamide | 75.9 ± 3.4 |  | 760 | L-3,4-Dihydroxyphenylalanine methyl ester hydrochloride | 98.6 ± 13.3 |
| 121 | 10058-F4 | 75.9 ± 8.5 |  | 761 | Naftopidil dihydrochloride | 98.6 ± 14.2 |
| 122 | WB-4101 hydrochloride | 76.0 ± 5.8 |  | 762 | (±)-threo-1-Phenyl-2-decanoylamino-3-morpholino-1-propanol hydrochloride | 98.6 ± 11.0 |
| 123 | SB-525334 | 76.3 ± 9.5 |  | 763 | S(+)-Raclopride L-tartrate | 98.6 ± 4.9 |
| 124 | alpha-Guanidinoglutaric acid | 76.4 ± 9.5 |  | 764 | Rolipram | 98.7 ± 10.9 |
| 125 | Olvanil | 76.7 ± 2.8 |  | 765 | Tropicamide | 98.7 ± 3.0 |
| 126 | SB 222200 | 76.8 ± 5.5 |  | 766 | Histamine, R(-)-alpha-methyl-, dihydrochloride | 98.7 ± 8.4 |
| 127 | FAUC 213 | 76.8 ± 3.4 |  | 767 | 5alpha-Pregnan-3alpha-ol-11,20-dione | 98.7 ± 9.4 |
| 128 | Betamethasone | 77.0 ± 7.6 |  | 768 | Felbamate | 98.7 ± 4.1 |
| 129 | L-798106 | 77.1 ± 10.4 |  | 769 | Nilutamide | 98.7 ± 10.0 |
| 130 | p-Iodoclonidine hydrochloride | 77.2 ± 42.2 |  | 770 | 4-Hydroxyphenethylamine hydrochloride | 98.7 ± 19.0 |
| 131 | CP-154526 hydrochloride | 77.3 ± 23.1 |  | 771 | N-(3,3-Diphenylpropyl)glycinamide | 98.7 ± 7.8 |
| 132 | Nelfinavir mesylate hydrate | 77.3 ± 17.3 |  | 772 | MK-886 | 98.7 ± 12.2 |
| 133 | TG003 | 77.3 ± 17.0 |  | 773 | Semicarbazide hydrochloride | 98.7 ± 22.6 |
| 134 | 6-Fluoronorepinephrine hydrochloride | 77.6 ± 28.4 |  | 774 | Ciprofibrate | 98.7 ± 5.2 |
| 135 | CP-64434 hydrate | 77.6 ± 21.9 |  | 775 | CP-471474 | 98.7 ± 17.5 |
| 136 | Hispidin | 77.8 ± 19.6 |  | 776 | Eliprodil | 98.8 ± 8.5 |
| 137 | R(+)-6-Bromo-APB hydrobromide | 77.8 ± 20.1 |  | 777 | 5-Fluorouracil | 98.8 ± 8.7 |
| 138 | 7-Chloro-4-hydroxy-2-phenyl-1,8-naphthyridine | 77.8 ± 8.8 |  | 778 | Ro 41-0960 | 98.8 ± 6.8 |
| 139 | GR 79236X | 78.0 ± 19.3 |  | 779 | Benazoline oxalate | 98.8 ± 14.3 |
| 140 | Ellipticine | 78.2 ± 23.8 |  | 780 | Tryptamine hydrochloride | 98.8 ± 5.7 |
| 141 | GYKI 52466 hydrochloride | 78.2 ± 10.4 |  | 781 | Dicyclomine hydrochloride | 98.9 ± 10.8 |
| 142 | Pimozide | 78.2 ± 10.9 |  | 782 | Supercinnamaldehyde | 98.9 ± 0.6 |
| 143 | Gallamine triethiodide | 78.3 ± 38.7 |  | 783 | Tracazolate | 98.9 ± 4.3 |
| 144 | BF-170 hydrochloride | 78.6 ± 20.5 |  | 784 | Azithromycin dihydrate | 98.9 ± 6.9 |
| 145 | Betaine hydrochloride | 78.7 ± 15.6 |  | 785 | Phentolamine mesylate | 98.9 ± 10.7 |
| 146 | Dipyridamole | 78.8 ± 14.8 |  | 786 | Tiapride hydrochloride | 98.9 ± 15.2 |
| 147 | Disopyramide | 78.9 ± 27.0 |  | 787 | 4-Amidinophenylmethanesulfonyl fluoride hydrochloride | 98.9 ± 8.1 |
| 148 | PNU-282987 | 78.9 ± 33.0 |  | 788 | Oleic Acid | 98.9 ± 11.8 |
| 149 | Nocodazole | 79.0 ± 5.7 |  | 789 | Bupropion hydrochloride | 98.9 ± 4.9 |
| 150 | Piceatannol | 79.1 ± 22.1 |  | 790 | Phosphomycin disodium | 98.9 ± 12.7 |
| 151 | L-165,041 | 79.1 ± 19.7 |  | 791 | Benserazide hydrochloride | 98.9 ± 5.3 |
| 152 | Felodipine | 79.1 ± 13.7 |  | 792 | Ketoconazole | 98.9 ± 9.0 |
| 153 | Cyclophosphamide monohydrate | 79.2 ± 18.9 |  | 793 | 2-Methylthioadenosine triphosphate tetrasodium | 99.0 ± 0.7 |
| 154 | Cefaclor | 79.3 ± 20.7 |  | 794 | Triflupromazine hydrochloride | 99.0 ± 16.0 |
| 155 | Caffeic acid phenethyl ester | 79.3 ± 10.7 |  | 795 | N-Acetyltryptamine | 99.0 ± 14.1 |
| 156 | Nordihydroguaiaretic acid from Larrea divaricata (creosote bush) | 79.3 ± 28.3 |  | 796 | Benzamide | 99.1 ± 4.7 |
| 157 | Ritanserin | 79.4 ± 12.5 |  | 797 | Moxonidine hydrochloride | 99.1 ± 3.2 |
| 158 | 8-(3-Chlorostyryl)caffeine | 79.6 ± 7.9 |  | 798 | L-3,4-Dihydroxyphenylalanine | 99.1 ± 9.5 |
| 159 | Loxapine succinate | 80.2 ± 12.7 |  | 799 | Theophylline | 99.1 ± 10.8 |
| 160 | Phorbol 12-myristate 13-acetate | 80.3 ± 5.6 |  | 800 | 3-(1H-Imidazol-4-yl)propyl di(p-fluorophenyl)methyl ether hydrochloride | 99.1 ± 1.1 |
| 161 | NU6027 | 80.6 ± 9.9 |  | 801 | Altretamine | 99.1 ± 9.2 |
| 162 | ET-18-OCH3 | 80.6 ± 4.5 |  | 802 | 8-Methoxymethyl-3-isobutyl-1-methylxanthine | 99.2 ± 10.1 |
| 163 | Promazine hydrochloride | 80.6 ± 8.4 |  | 803 | Formoterol | 99.2 ± 6.5 |
| 164 | erythro-9-(2-Hydroxy-3-nonyl)adenine hydrochloride | 80.7 ± 17.0 |  | 804 | Aminoguanidine hemisulfate | 99.2 ± 8.4 |
| 165 | PD 98,059 | 80.7 ± 2.6 |  | 805 | Diethylenetriaminepentaacetic acid | 99.2 ± 10.9 |
| 166 | Gabapentin | 80.7 ± 22.8 |  | 806 | Imipramine hydrochloride | 99.2 ± 4.6 |
| 167 | Debrisoquin sulfate | 81.0 ± 16.3 |  | 807 | (±)-Chlorpheniramine maleate | 99.2 ± 9.9 |
| 168 | Phenserine | 81.1 ± 29.6 |  | 808 | PF-4708671 | 99.2 ± 13.5 |
| 169 | 3-Bromo-7-nitroindazole | 81.2 ± 6.7 |  | 809 | Dihydroergotamine methanesulfonate | 99.2 ± 11.1 |
| 170 | CGP 57380 | 81.2 ± 23.0 |  | 810 | (±)-6-Chloro-PB hydrobromide | 99.3 ± 4.1 |
| 171 | Fenspiride hydrochloride | 81.2 ± 5.0 |  | 811 | Hydroxylamine hydrochloride | 99.3 ± 3.4 |
| 172 | cDPCP | 81.3 ± 5.4 |  | 812 | Guvacine hydrochloride | 99.3 ± 14.1 |
| 173 | Clofibrate | 81.3 ± 24.9 |  | 813 | (-)-Quinpirole hydrochloride | 99.3 ± 13.8 |
| 174 | Esomeprazole magnesium dihydrate | 81.5 ± 16.8 |  | 814 | 2,3-Dimethoxy-1,4-naphthoquinone | 99.3 ± 8.8 |
| 175 | Tyrphostin 1 | 81.6 ± 2.9 |  | 815 | (-)-Physostigmine | 99.3 ± 6.1 |
| 176 | SB 200646 hydrochloride | 81.7 ± 19.9 |  | 816 | Imidazole-4-acetic acid hydrochloride | 99.3 ± 14.9 |
| 177 | Arecoline hydrobromide | 81.8 ± 27.0 |  | 817 | L-Aspartic acid | 99.3 ± 3.7 |
| 178 | N-Succinyl-L-proline | 81.8 ± 11.2 |  | 818 | CP-335963 | 99.3 ± 11.5 |
| 179 | Staurosporine aglycone | 81.9 ± 6.1 |  | 819 | Mexiletene hydrochloride | 99.4 ± 6.9 |
| 180 | Pentoxifylline | 81.9 ± 17.4 |  | 820 | Ritodrine hydrochloride | 99.4 ± 8.5 |
| 181 | AMN082 | 81.87813367 |  | 821 | (±)-cis-Piperidine-2,3-dicarboxylic acid | 99.4 ± 1.9 |
| 182 | Fenoterol hydrobromide | 81.9 ± 17.1 |  | 822 | Trihexyphenidyl hydrochloride | 99.4 ± 5.7 |
| 183 | Fenobam | 81.9 ± 15.0 |  | 823 | Artemether | 99.4 ± 9.9 |
| 184 | Auranofin | 82.1 ± 31.0 |  | 824 | (±)-SKF-38393 hydrochloride | 99.4 ± 9.0 |
| 185 | SANT-1 | 82.1 ± 15.9 |  | 825 | Hexamethonium bromide | 99.4 ± 1.1 |
| 186 | 2',3'-didehydro-3'-deoxythymidine | 82.1 ± 11.9 |  | 826 | Phenelzine sulfate | 99.4 ± 7.6 |
| 187 | Ro 04-6790 dihydrochloride | 82.1 ± 15.5 |  | 827 | N-Methylhistaprodifen dioxalate salt | 99.4 ± 7.3 |
| 188 | 3'-Azido-3'-deoxythymidine | 82.3 ± 18.1 |  | 828 | S-(+)-PD 123177 trifluoroacetate salt hydrate | 99.4 ± 7.4 |
| 189 | S-(p-Azidophenacyl)glutathione | 82.4 ± 8.6 |  | 829 | AIDA | 99.4 ± 5.4 |
| 190 | Wortmannin from Penicillium funiculosum | 82.6 ± 17.7 |  | 830 | Clomipramine hydrochloride | 99.4 ± 3.8 |
| 191 | BRL 50481 | 82.8 ± 17.4 |  | 831 | Lorglumide sodium | 99.4 ± 5.5 |
| 192 | BMY 7378 dihydrochloride | 82.8 ± 23.0 |  | 832 | (+)-Norfenfluramine hydrochloride | 99.5 ± 11.3 |
| 193 | Pergolide methanesulfonate | 82.8 ± 15.4 |  | 833 | S-Nitrosoglutathione | 99.5 ± 12.8 |
| 194 | Ibudilast | 82.8 ± 12.0 |  | 834 | 8-Bromo-cAMP sodium | 99.5 ± 9.1 |
| 195 | Palmitoyl-DL-Carnitine chloride | 82.9 ± 21.8 |  | 835 | Flumazenil | 99.5 ± 3.5 |
| 196 | Lercanidipine hydrochloride hemihydrate | 82.9 ± 16.7 |  | 836 | NCS-382 | 99.5 ± 31.9 |
| 197 | R(-)-2,10,11-Trihydroxyaporphine hybrobromide | 83.0 ± 14.1 |  | 837 | O-(Carboxymethyl)hydroxylamine hemihydrochloride | 99.5 ± 10.8 |
| 198 | MRS 2159 | 83.0 ± 11.6 |  | 838 | Domperidone | 99.6 ± 10.5 |
| 199 | R-(+)-8-Hydroxy-DPAT hydrobromide | 83.0 ± 17.5 |  | 839 | DL-Homatropine hydrobromide | 99.6 ± 8.1 |
| 200 | Tamoxifen | 83.1 ± 23.3 |  | 840 | (±)-Baclofen | 99.6 ± 6.9 |
| 201 | (±)-Octoclothepin maleate | 83.2 ± 25.2 |  | 841 | Sandoz 58-035 | 99.6 ± 12.7 |
| 202 | L-701,324 | 83.2 ± 4.8 |  | 842 | (S)-(+)-Camptothecin | 99.7 ± 12.1 |
| 203 | Clozapine | 83.2 ± 22.9 |  | 843 | TPMPA | 99.7 ± 3.7 |
| 204 | SC-57461A | 83.2 ± 13.1 |  | 844 | Clemizole hydrochloride | 99.7 ± 8.2 |
| 205 | (±)-Metoprolol (+)-tartrate | 83.3 ± 9.2 |  | 845 | (±)-SKF 38393, N-allyl-, hydrobromide | 99.7 ± 11.6 |
| 206 | AS605240 | 83.4 ± 13.4 |  | 846 | (±)-alpha-Lipoic Acid | 99.7 ± 5.6 |
| 207 | SCH-202676 hydrobromide | 83.4 ± 13.1 |  | 847 | Trandolapril | 99.7 ± 12.5 |
| 208 | CPNQ | 83.4 ± 4.6 |  | 848 | Trimethoprim | 99.7 ± 13.3 |
| 209 | 1-Aminobenzotriazole | 83.5 ± 18.8 |  | 849 | (-)-Scopolamine hydrobromide | 99.7 ± 17.0 |
| 210 | Kynurenic acid | 83.5 ± 10.7 |  | 850 | Thioperamide maleate | 99.7 ± 10.4 |
| 211 | Urapidil, 5-Methyl- | 83.5 ± 22.8 |  | 851 | 1-Methylhistamine dihydrochloride | 99.7 ± 10.5 |
| 212 | Mifepristone | 83.5 ± 5.2 |  | 852 | Allopurinol | 99.8 ± 7.2 |
| 213 | CP-226269 | 83.6 ± 17.8 |  | 853 | Corticosterone | 99.8 ± 5.3 |
| 214 | Ganaxolone | 83.6 ± 13.4 |  | 854 | N-Ethylmaleimide | 99.8 ± 13.3 |
| 215 | Amitriptyline hydrochloride | 83.6 ± 19.8 |  | 855 | (-)-cis-(1S,2R)-U-50488 tartrate | 99.8 ± 10.5 |
| 216 | AC-55649 | 83.6 ± 13.2 |  | 856 | Valproic acid sodium | 99.8 ± 9.6 |
| 217 | trans-(±)-ACPD | 83.6 ± 13.4 |  | 857 | Doxazosin mesylate | 99.9 ± 9.5 |
| 218 | L-Cysteinesulfinic Acid | 83.6 ± 21.3 |  | 858 | Amsacrine hydrochloride | 99.8 ± 8.3 |
| 219 | BAY 61-3606 hydrochloride hydrate | 83.8 ± 10.8 |  | 859 | (±)-2-Amino-4-phosphonobutyric acid | 99.9 ± 8.9 |
| 220 | Reserpine | 83.8 ± 14.9 |  | 860 | Imetit dihydrobromide | 99.9 ± 2.2 |
| 221 | NF 023 | 83.8 ± 25.8 |  | 861 | Tulobuterol hydrochloride | 99.9 ± 4.7 |
| 222 | K 185 | 84.1 ± 1.4 |  | 862 | U-73343 | 99.9 ± 5.3 |
| 223 | Hydrocortisone | 84.4 ± 24.1 |  | 863 | Acyclovir | 99.9 ±13.7 |
| 224 | Flutamide | 84.4 ± 23.0 |  | 864 | BTO-1 | 99.9 ± 23.7 |
| 225 | Sulindac sulfone | 84.5 ± 13.2 |  | 865 | L-Glutamine | 99.9 ± 14.1 |
| 226 | Pyrilamine maleate | 84.6 ± 34.1 |  | 866 | Lithium Chloride | 99.9 ± 5.2 |
| 227 | JX401 | 84.6 ± 8.9 |  | 867 | Diclofenac sodium | 100.0 ± 12.4 |
| 228 | Cefmetazole sodium | 84.6 ± 22.3 |  | 868 | DL-Thiorphan | 100.0 ± 12.5 |
| 229 | Pindolol | 84.7 ± 11.8 |  | 869 | Quipazine, 6-nitro-, maleate | 100.0 ± 4.7 |
| 230 | Ziprasidone hydrochloride monohydrate | 84.7 ± 3.4 |  | 870 | Choline bromide | 100.0 ± 6.6 |
| 231 | Chlormethiazole hydrochloride | 84.7 ± 21.7 |  | 871 | L-Tryptophan | 100.0 ± 3.7 |
| 232 | N-Methyl-beta-carboline-3-carboxamide | 84.9 ± 8.4 |  | 872 | 3,5-Dinitrocatechol | 100.0 ± 11.8 |
| 233 | 4-DAMP methiodide | 84.9 ± 19.8 |  | 873 | SKF 96365 | 100.0 ± 16.7 |
| 234 | Tyrphostin 23 | 84.9 ± 6.4 |  | 874 | AFMK | 100.0 ± 2.7 |
| 235 | Loratadine | 85.0 ± 1.8 |  | 875 | Caffeic Acid | 100.0 ± 4.2 |
| 236 | SB 415286 | 85.0 ± 3.4 |  | 876 | R(-)-Me5 | 100.1 ± 8.0 |
| 237 | DNQX | 85.0 ± 6.8 |  | 877 | Leflunomide | 100.1 ± 5.3 |
| 238 | Spiperone hydrochloride | 85.1 ± 18.3 |  | 878 | Methotrexate hydrate | 100.1 ± 8.9 |
| 239 | 5alpha-Pregnan-3alpha-ol-20-one | 85.2 ± 19.4 |  | 879 | Tranylcypromine hydrochloride | 100.1 ± 11.2 |
| 240 | 13-cis-retinoic acid | 85.2 ± 2.9 |  | 880 | Ketanserin tartrate | 100.1 ± 15.2 |
| 241 | Cyclobenzaprine hydrochloride | 85.3 ± 24.5 |  | 881 | Avridine | 100.2 ± 21.9 |
| 242 | 5'-Amino-5'-deoxyadenosine p-toluenesulfonate salt | 85.5 ± 26.7 |  | 882 | Neostigmine bromide | 100.2 ± 23.4 |
| 243 | 5-Carboxamidotryptamine maleate | 85.7 ± 14.5 |  | 883 | NS 2028 | 100.2 ± 8.6 |
| 244 | Tetracaine hydrochloride | 85.8 ± 26.7 |  | 884 | (S)-Propranolol hydrochloride | 100.2 ± 8.9 |
| 245 | p-Benzoquinone | 85.9 ± 13.7 |  | 885 | 9-Amino-1,2,3,4-tetrahydroacridine hydrochloride | 100.2 ± 15.6 |
| 246 | (R,R)-cis-Diethyl tetrahydro-2,8-chrysenediol | 85.9 ± 6.0 |  | 886 | D-Serine | 100.2 ± 11.0 |
| 247 | Dequalinium chloride hydrate | 86.1 ± 10.0 |  | 887 | THIP hydrochloride | 100.3 ± 3.4 |
| 248 | Etoposide | 86.36471682 |  | 888 | PRE-084 | 100.3 ± 14.3 |
| 249 | SMER28 | 86.4 ± 8.7 |  | 889 | Lansoprazole | 100.3 ± 13.0 |
| 250 | N-Acetylprocainamide hydrochloride | 86.4 ± 17.7 |  | 890 | Resveratrol | 100.3 ± 16.3 |
| 251 | Danazol | 86.4 ± 9.1 |  | 891 | Ketoprofen | 100.3 ± 5.0 |
| 252 | Papaverine hydrochloride | 86.6 ± 22.0 |  | 892 | 7,7-Dimethyl-(5Z,8Z)-eicosadienoic acid | 100.3 ± 12.4 |
| 253 | Dihydrocapsaicin | 86.7 ± 9.6 |  | 893 | 2-(Methylthio)adenosine 5'-diphosphate trisodium salt hydrate | 100.3 ± 5.3 |
| 254 | (±)-3-(3,4-dihydroxyphenyl)-2-methyl-DL-alanine | 86.8 ± 7.1 |  | 894 | N-Acetyl-L-Cysteine | 100.3 ± 0.2 |
| 255 | Biperiden hydrochloride | 86.9 ± 6.4 |  | 895 | Pentamidine isethionate | 100.3 ± 6.3 |
| 256 | Cephalosporin C zinc salt | 86.9 ± 28.2 |  | 896 | Fulvestrant | 100.4 ± 12.1 |
| 257 | SC-51322 | 86.9 ± 5.7 |  | 897 | (-)-alpha-Methylnorepinephrine | 100.4 ± 6.1 |
| 258 | SDZ-205,557 hydrochloride | 87.0 ± 9.3 |  | 898 | PPNDS tetrasodium | 100.4 ± 12.5 |
| 259 | Me-3,4-dephostatin | 87.0 ± 11.0 |  | 899 | L-Histidine hydrochloride | 100.4 ± 6.4 |
| 260 | CBIQ | 87.1 ± 7.0 |  | 900 | (±)-2,3-Dichloro-alpha-methylbenzylamine hydrochloride | 100.4 ± 9.9 |
| 261 | (±)-Norepinephrine (+)bitartrate | 87.1 ± 8.1 |  | 901 | 6,7-ADTN hydrobromide | 100.4 ± 8.1 |
| 262 | 1-(4-Hydroxybenzyl)imidazole-2-thiol | 87.3 ± 21.6 |  | 902 | Phenamil methanesulfonate | 100.4 ± 3.8 |
| 263 | A-77636 hydrochloride | 87.3 ± 8.5 |  | 903 | Granisetron hydrochloride | 100.4 ± 14.5 |
| 264 | Isoguvacine hydrochloride | 87.4 ± 16.5 |  | 904 | N-Acetyl-5-hydroxytryptamine | 100.5 ± 7.6 |
| 265 | Brefeldin A from Penicillium brefeldianum | 87.5 ± 5.2 |  | 905 | Opipramol dihydrochloride | 100.5 ± 7.4 |
| 266 | 5-(N,N-hexamethylene)amiloride | 87.5 ± 4.4 |  | 906 | (-)-Epinephrine bitartrate | 100.5 ± 6.3 |
| 267 | CP-91149 | 87.6 ± 14.6 |  | 907 | Linezolid | 100.5 ± 2.1 |
| 268 | Fenofibrate | 87.6 ± 17.1 |  | 908 | Praziquantel | 100.5 ± 15.0 |
| 269 | CGP-7930 | 87.6 ± 4.9 |  | 909 | Ceftriaxone sodium | 100.5 ± 11.9 |
| 270 | XCT790 | 87.7 ± 9.0 |  | 910 | Hydralazine hydrochloride | 100.6 ± 8.6 |
| 271 | beta-Estradiol | 87.8 ± 13.4 |  | 911 | (±)-AMT hydrochloride | 100.6 ± 12.5 |
| 272 | 1-(2-Chlorophenyl)-1-(4-chlorophenyl)-2,2-dichloroethane | 87.8 ± 23.1 |  | 912 | L-655,708 | 100.6 ± 12.8 |
| 273 | DCEBIO | 87.8 ± 8.9 |  | 913 | Uridine 5'-diphosphate sodium | 100.6 ± 5.7 |
| 274 | Isoliquiritigenin | 88.0 ± 9.1 |  | 914 | Yohimbine hydrochloride | 100.6 ± 0.5 |
| 275 | CP-380736 | 88.0 ± 4.4 |  | 915 | Hydroquinone | 100.7 ± 6.6 |
| 276 | SB 204741 | 88.1 ± 14.4 |  | 916 | E-64 | 100.7 ± 6.0 |
| 277 | Sildenafil citrate salt | 88.1 ± 8.0 |  | 917 | Olprinone hydrochloride | 100.7 ± 4.5 |
| 278 | Edrophonium chloride | 88.1 ± 20.0 |  | 918 | L-azetidine-2-carboxylic acid | 100.7 ± 4.1 |
| 279 | Tetraethylthiuram disulfide | 88.1 ± 9.2 |  | 919 | N-Methyl-1-deoxynojirimycin | 100.7 ± 20.9 |
| 280 | Doxycycline hydrochloride | 88. 2 ± 13.0 |  | 920 | Hexamethonium dichloride | 100.8 ± 9.2 |
| 281 | Trequinsin hydrochloride | 88.3 ± 40.0 |  | 921 | BU224 hydrochloride | 100.8 ± 7.2 |
| 282 | 1-Aminocyclopropanecarboxylic acid hydrochloride | 88.3 ± 14.5 |  | 922 | Z-L-Phe chloromethyl ketone | 100.8 ± 7.1 |
| 283 | CPCCOEt | 88.3 ± 3.9 |  | 923 | Carvedilol | 100.8 ± 14.2 |
| 284 | Ethosuximide | 88.4 ± 5.5 |  | 924 | Iofetamine hydrochloride | 100.8 ± 7.1 |
| 285 | R(+)-3PPP hydrochloride | 88.4 ± 12.1 |  | 925 | Vancomycin hydrochloride from Streptomyces orientalis | 100.8 ± 8.6 |
| 286 | Tyrphostin AG 698 | 88.4 ± 5.8 |  | 926 | Cefsulodin sodium salt hydrate | 100.8 ± 6.6 |
| 287 | SIB 1893 | 88.4 ± 9.6 |  | 927 | 1,7-Dimethylxanthine | 100.9 ± 19.4 |
| 288 | Icilin | 88.5 ± 5.8 |  | 928 | Forskolin | 100.9 ± 7.2 |
| 289 | N,N-Dihexyl-2-(4-fluorophenyl)indole-3-acetamide | 88.5 ± 10.2 |  | 929 | BW 284c51 | 100.9 ± 3.8 |
| 290 | Isonipecotic acid | 88.5 ± 19.5 |  | 930 | Rilmenidine hemifumarate | 100.9 ± 6.4 |
| 291 | Amiloride hydrochloride | 88.5 ± 23.0 |  | 931 | 5,7-Dichlorokynurenic acid | 100.9 ± 8.8 |
| 292 | Mitoxantrone | 88.5 ± 12.7 |  | 932 | Rufinamide | 100.9 ± 12.8 |
| 293 | (-)-Scopolamine methyl bromide | 88.5 ± 14.0 |  | 933 | Aminoguanidine hydrochloride | 100.9 ± 6.1 |
| 294 | Pirenperone | 88.6 ± 5.4 |  | 934 | GR 46611 | 100.9 ± 13.0 |
| 295 | Dofetilide | 88.8 ± 14.7 |  | 935 | Pregnenolone sulfate sodium | 100.9 ± 23.1 |
| 296 | Perphenazine | 88.8 ± 23.6 |  | 936 | Fluvoxamine maleate | 100.9 ± 16.7 |
| 297 | Nefiracetam | 88.8 ± 23.8 |  | 937 | 3-n-Propylxanthine | 101.0 ± 10.1 |
| 298 | IC 261 | 88.8 ± 5.2 |  | 938 | R-(-)-Desmethyldeprenyl hydrochloride | 101.0 ± 11.7 |
| 299 | Daidzein | 88.9 ± 6.0 |  | 939 | Cephalexin hydrate | 101.0 ± 8.0 |
| 300 | Pyrazinecarboxamide | 88.9 ± 26.1 |  | 940 | Propionylpromazine hydrochloride | 101.0 ± 0.8 |
| 301 | p-Aminoclonidine hydrochloride | 89.0 ± 23.3 |  | 941 | RX 821002 hydrochloride | 101.0 ± 17.5 |
| 302 | R(-)-2,10,11-Trihydroxy-N-propylnoraporphine hydrobromide | 89.0 ± 14.4 |  | 942 | Piroxicam | 101.0 ± 12.2 |
| 303 | (S)-MAP4 hydrochloride | 89.0 ± 15.4 |  | 943 | Oxybutynin Chloride | 101.0 ± 12.8 |
| 304 | Alloxazine | 89.0 ± 4.9 |  | 944 | Sertraline hydrochloride | 101.1 ± 11.7 |
| 305 | DPO-1 | 89.1 ± 3.8 |  | 945 | L-Canavanine | 101.1 ± 4.7 |
| 306 | Orphenadrine hydrochloride | 89.1 ± 18.3 |  | 946 | Oxolinic acid | 101.1 ± 12.4 |
| 307 | Sulfaphenazole | 89.1 ± 34.9 |  | 947 | S(+)-Isoproterenol (+)-bitartrate | 101.2 ± 2.4 |
| 308 | Aminophylline ethylenediamine | 89.2 ± 8.4 |  | 948 | 1-[2-(Trifluoromethyl)phenyl]imidazole | 101.2 ± 9.4 |
| 309 | Cantharidin | 89.3 ± 25.6 |  | 949 | N^G,N^G-Dimethylarginine hydrochloride | 101.2 ± 10.7 |
| 310 | Cysteamine hydrochloride | 89.3 ± 6.0 |  | 950 | P1,P4-Di(adenosine-5')tetraphosphate triammonium | 101.2 ± 10.2 |
| 311 | L-Glutamic acid, N-phthaloyl- | 89.4 ± 32.8 |  | 951 | Droperidol | 101.2 ± 18.5 |
| 312 | CI-976 | 89.4 ± 17.7 |  | 952 | Phosphoramidon disodium | 101.2 ± 7.1 |
| 313 | 2-Chloroadenosine triphosphate tetrasodium | 89.5 ± 23.0 |  | 953 | Tetradecylthioacetic acid | 101.3 ± 12.1 |
| 314 | (-)-Scopolamine methyl nitrate | 89.5 ± 24.4 |  | 954 | 2,3-Butanedione | 101.3 ± 15.0 |
| 315 | Procainamide hydrochloride | 89.6 ± 33.7 |  | 955 | U-99194A maleate | 101.3 ± 8.9 |
| 316 | NBI 27914 | 89.7 ± 7.0 |  | 956 | S-(-)-Carbidopa | 101.3 ± 7.8 |
| 317 | Carbamazepine | 89.8 ± 14.0 |  | 957 | Oxotremorine methiodide | 101.3 ± 18.6 |
| 318 | 2-Chloro-2-deoxy-D-glucose | 89.9 ± 5.0 |  | 958 | Thio-NADP sodium | 101.4 ± 10.4 |
| 319 | Furegrelate sodium | 89.9 ± 4.6 |  | 959 | Chlormezanone | 101.4 ± 10.9 |
| 320 | AC-93253 iodide | 89.8 ± 11.3 |  | 960 | Acetohexamide | 101.4 ± 13.1 |
| 321 | 3-Aminopropylphosphonic acid | 90.0 ± 2.1 |  | 961 | 4-Imidazolemethanol hydrochloride | 101.5 ± 8.2 |
| 322 | 1,4-Dideoxy-1,4-imino-D-arabinitol | 90.0 ± 6.2 |  | 962 | (±)-Brompheniramine maleate | 101.5 ± 4.3 |
| 323 | SKF 89626 | 90.14026278 |  | 963 | L-2-aminoadipic acid | 101.5 ± 9.7 |
| 324 | Tyrphostin AG 538 | 90.2 ± 14.0 |  | 964 | (E)-4-amino-2-butenoic acid | 101.5 ± 12.6 |
| 325 | Triprolidine hydrochloride | 90.2 ± 0.5 |  | 965 | Chlorzoxazone | 101.5 ± 2.4 |
| 326 | Tyrphostin AG 1478 | 90.3 ± 8.9 |  | 966 | Diazoxide | 101.5 ± 12.2 |
| 327 | alpha-Lobeline hydrochloride | 90.3 ± 6.6 |  | 967 | Protriptyline hydrochloride | 101.6 ± 7.3 |
| 328 | Centrophenoxine hydrochloride | 90.4 ± 28.8 |  | 968 | Mizoribine | 101.6 ± 4.8 |
| 329 | Prochlorperazine dimaleate | 90.4 ± 18.3 |  | 969 | MDL 105,519 | 101.6 ± 19.9 |
| 330 | Varenicline tartrate | 90.5 ± 2.6 |  | 970 | Niclosamide | 101.6 ± 22.5 |
| 331 | Metolazone | 90.5 ± 39.1 |  | 971 | 5-Bromo-2'-deoxyuridine | 101.6 ± 8.3 |
| 332 | B-HT 933 dihydrochloride | 90.5 ± 9.0 |  | 972 | (6R)-5,6,7,8-Tetrahydro-L-biopterin hydrochloride | 101.6 ± 17.7 |
| 333 | Capsazepine | 90.6 ± 11.8 |  | 973 | Theobromine | 101.7 ± 4.5 |
| 334 | Fenoldopam bromide | 90.6 ± 5.5 |  | 974 | (±)-PPHT hydrochloride | 101.7 ± 5.1 |
| 335 | (±)-Synephrine | 90.7 ± 28.9 |  | 975 | Vanillic acid diethylamide | 101.7 ± 2.4 |
| 336 | PD-161570 | 90.7 ± 9.1 |  | 976 | Minocycline hydrochloride | 101.7 ± 17.9 |
| 337 | 1,10-Phenanthroline monohydrate | 90.7 ± 13.0 |  | 977 | Bepridil hydrochloride | 101.8 ± 6.3 |
| 338 | Acepromazine maleate | 90.7 ± 4.5 |  | 978 | Diphenhydramine hydrochloride | 101.8 ± 15.9 |
| 339 | (±)-2-Amino-5-phosphonopentanoic acid | 90.8 ± 6.2 |  | 979 | Tolbutamide | 101.8 ± 7.4 |
| 340 | WAY-100635 maleate | 90.8 ± 17.8 |  | 980 | Dipropyldopamine hydrobromide | 101.8 ± 8.0 |
| 341 | Atropine methyl nitrate | 90.8 ± 8.8 |  | 981 | Dobutamine hydrochloride | 101.9 ± 15.9 |
| 342 | Benzamidine hydrochloride | 90.8 ± 8.3 |  | 982 | (±)-Nipecotic acid | 101.9 ± 5.9 |
| 343 | Raloxifene hydrochloride | 90.8 ± 14.0 |  | 983 | Oxotremorine sesquifumarate salt | 101.9 ± 13.3 |
| 344 | SC-236 | 90.8 ± 15.2 |  | 984 | Iodoacetamide | 101.9 ± 8.2 |
| 345 | Estrone | 91.0 ± 8.5 |  | 985 | ABT-418 hydrochloride | 101.9 ± 4.7 |
| 346 | Kainic acid | 90.1 ± 18.1 |  | 986 | L-Hyoscyamine | 101.9 ± 12.00 |
| 347 | Pyrocatechol | 91.0 ± 7.4 |  | 987 | Clonidine hydrochloride | 101.9 ± 9.8 |
| 348 | N-(4-Amino-2-chlorophenyl)phthalimide | 91.1 ± 7.1 |  | 988 | Terfenadine | 101.9 ± 5.3 |
| 349 | Aminopterin | 91.1 ± 3.5 |  | 989 | Ouabain | 102.0 ± 3.5 |
| 350 | 5HPP-33 | 91.1 ± 9.2 |  | 990 | Tocainide hydrochloride | 102.0 ± 15.0 |
| 351 | NAN-190 hydrobromide | 91.1 ± 29.0 |  | 991 | S-Methyl-L-thiocitrulline acetate | 102.0 ± 13.0 |
| 352 | L-732,138 | 91.1 ± 5.0 |  | 992 | S-(+)-Fluoxetine hydrochloride | 102.0 ± 9.9 |
| 353 | R(+)-Butylindazone | 91.2 ± 11.8 |  | 993 | N-p-Tosyl-L-phenylalanine chloromethyl ketone | 102.0 ± 13.7 |
| 354 | ML-9 | 91.2 ± 7.1 |  | 994 | Histamine dihydrochloride | 102.0 ± 10.2 |
| 355 | Molindone hydrochloride | 91.2 ± 7.2 |  | 995 | Daphnetin | 102.0 ± 13.1 |
| 356 | NS8593 hydrochloride | 91.2 ± 11.5 |  | 996 | Dextromethorphan hydrobromide monohydrate | 102.0 ± 12.4 |
| 357 | Tetrabenazine | 91.3 ± 18.4 |  | 997 | Metaproterenol hemisulfate | 102.0 ± 5.4 |
| 358 | Acetyl-beta-methylcholine chloride | 91.4 ± 19.2 |  | 998 | Topotecan hydrochloride hydrate | 102.0 ± 7.8 |
| 359 | (±)-Ibuprofen | 91.4 ± 12.3 |  | 999 | Isotharine mesylate | 102.1 ± 6.6 |
| 360 | Tyrphostin AG 494 | 91.5 ± 7.4 |  | 1000 | (±)-Sulpiride | 102.1 ± 6.1 |
| 361 | Pheniramine maleate | 91.5 ± 14.9 |  | 1001 | U-101958 maleate | 102.1 ± 9.0 |
| 362 | S-Ethylisothiourea hydrobromide | 91.5 ± 10.5 |  | 1002 | UK 14,304 | 102.1 ± 10.4 |
| 363 | 2-(2-Aminoethyl)isothiourea dihydrobromide | 91.5 ± 12.4 |  | 1003 | Flunarizine dihydrochloride | 102.1 ± 8.8 |
| 364 | Amiodarone hydrochloride | 91.5 ± 10.5 |  | 1004 | CP-93129 dihydrochloride hydrate | 102.1 ± 6.9 |
| 365 | 3-aminobenzamide | 91.6 ± 2.5 |  | 1005 | Ranitidine hydrochloride | 102.1 ± 11.6 |
| 366 | Methylergonovine maleate | 91.6 ± 8.0 |  | 1006 | Levetiracetam | 102.2 ± 9.6 |
| 367 | Azelaic acid | 91.8 ± 5.2 |  | 1007 | Phenylephrine hydrochloride | 102.2 ± 12.7 |
| 368 | Molsidomine | 91.8 ± 17.4 |  | 1008 | Spermidine trihydrochloride | 102.2 ± 19.0 |
| 369 | 8-(4-Chlorophenylthio)-cAMP sodium | 91.8 ± 2.6 |  | 1009 | Carmustine | 102.2 ± 1.0 |
| 370 | 1,3-Dimethyl-8-phenylxanthine | 91.8 ± 4.8 |  | 1010 | BW 723C86 | 102.2 ± 11.4 |
| 371 | 3-Aminopropionitrile fumarate | 91.9 ± 6.3 |  | 1011 | Atropine methyl bromide | 102.2 ± 7.3 |
| 372 | S-(4-Nitrobenzyl)-6-thioguanosine | 91.9 ± 3.2 |  | 1012 | 9-cyclopentyladenine | 102.2 ± 2.7 |
| 373 | Mianserin hydrochloride | 92.0 ± 7.7 |  | 1013 | 5-Hydroxyindolacetic acid | 102.3 ± 8.2 |
| 374 | Pyridostigmine bromide | 92.0 ± 6.7 |  | 1014 | CNS-1102 | 102.3 ± 11.4 |
| 375 | SB-366791 | 92.0 ± 7.1 |  | 1015 | Enoximone | 102.3 ± 5.2 |
| 376 | 5-azacytidine | 92.0 ± 10.7 |  | 1016 | alpha,beta-Methylene adenosine 5'-triphosphate dilithium | 102.3 ± 5.3 |
| 377 | Cortisone 21-acetate | 92.1 ± 5.7 |  | 1017 | Alfuzosin hydrochloride | 102.3 ± 4.9 |
| 378 | ML-7 | 92.1 ± 9.6 |  | 1018 | 4-Methylpyrazole hydrochloride | 102.3 ± 3.8 |
| 379 | Chlorpromazine hydrochloride | 92.2 ± 10.3 |  | 1019 | Cinnarizine | 102.3 ± 5.4 |
| 380 | Adenosine | 92.3 ± 7.9 |  | 1020 | Ranolazine dihydrochloride | 102.3 ± 13.7 |
| 381 | Pifithrin-mu | 92.3 ± 34.4 |  | 1021 | CP-101537 | 102.3 ± 6.7 |
| 382 | Methysergide maleate | 92.3 ± 21.2 |  | 1022 | 8-Bromo-cGMP sodium | 102.4 ± 7.4 |
| 383 | Rotenone | 92.4 ± 12.1 |  | 1023 | DL-alpha-Methyl-p-tyrosine | 102.4 ± 5.8 |
| 384 | Stevioside | 92.4 ± 13.9 |  | 1024 | Lidocaine hydrochloride | 102.4 ± 8.9 |
| 385 | Acetazolamide | 92.4 ± 4.0 |  | 1025 | Dihydroouabain | 102.4 ± 11.9 |
| 386 | PD 168,077 maleate | 92.4 ± 5.3 |  | 1026 | Ciproxifan hydrochloride | 102.4 ± 6.2 |
| 387 | Dihydrokainic acid | 92.4 ± 30.0 |  | 1027 | Thioridazine hydrochloride | 102.5 ± 9.6 |
| 388 | BWB70C | 92.4 ± 13.0 |  | 1028 | Cytidine 5'-diphosphocholine sodium salt hydrate | 102.5 ± 15.7 |
| 389 | 5-Fluoroindole-2-carboxylic acid | 92.5 ± 8.9 |  | 1029 | Acetylsalicylic acid | 102.5 ± 9.1 |
| 390 | Zimelidine dihydrochloride | 92.5 ± 9.4 |  | 1030 | Amoxapine | 102.6 ± 1.7 |
| 391 | Fiduxosin hydrochloride | 92.6 ± 13.4 |  | 1031 | Naltrexone hydrochloride | 102.6 ± 6.5 |
| 392 | L-alpha-Methyl DOPA | 92.6 ± 21.6 |  | 1032 | 1,1-Dimethyl-4-phenyl-piperazinium iodide | 102.6 ± 13.3 |
| 393 | Salmeterol xinafoate | 92.6 ± 8.3 |  | 1033 | (S)-3,5-Dihydroxyphenylglycine | 102.7 ± 20.4 |
| 394 | A-315456 | 92.6 ± 5.2 |  | 1034 | Emetine dihydrochloride hydrate | 102.7 ± 9.1 |
| 395 | Diphenyleneiodonium chloride | 92.7 ± 5.6 |  | 1035 | SQ 22536 | 102.7 ± 4.9 |
| 396 | Aminobenztropine | 92.7 ± 5.2 |  | 1036 | Terbutaline hemisulfate | 102.8 ± 10.1 |
| 397 | 2-Hydroxysaclofen | 92.7 ± 0.2 |  | 1037 | Tyrphostin AG 112 | 102.8 ± 4.7 |
| 398 | Budesonide | 92.7 ± 8.6 |  | 1038 | Trifluperidol hydrochloride | 102.8 ± 9.3 |
| 399 | Glybenclamide | 92.8 ± 16.7 |  | 1039 | MHPG sulfate potassium | 102.8 ± 6.8 |
| 400 | GR 113808 | 92.8 ± 8.0 |  | 1040 | BRL 54443 maleate | 102.8 ± 6.2 |
| 401 | 6-Chloromelatonin | 92.8 ± 24.3 |  | 1041 | Pargyline hydrochloride | 102.9 ± 12.8 |
| 402 | GR 55562 dihydrobromide | 92.8 ± 13.3 |  | 1042 | Bromoacetyl alprenolol menthane | 102.8 ± 4.8 |
| 403 | Pilocarpine nitrate | 92.9 ± 2.4 |  | 1043 | Naratriptan hydrochloride | 102.9 ± 17.6 |
| 404 | TTNPB | 92.9 ± 11.5 |  | 1044 | Fluoxetine hydrochloride | 102.9 ± 7.1 |
| 405 | N6-Cyclohexyladenosine | 92.9 ± 12.9 |  | 1045 | 1,3-Dipropyl-7-methylxanthine | 102.9 ± 21.6 |
| 406 | Amperozide hydrochloride | 92.9 ± 0.6 |  | 1046 | Buspirone hydrochloride | 102.9 ± 6.0 |
| 407 | Dopamine hydrochloride | 93.0 ± 11.1 |  | 1047 | Epibestatin hydrochloride | 102.9 ± 13.0 |
| 408 | ODQ | 93.0 ± 5.7 |  | 1048 | cis-4-Aminocrotonic acid | 103.0 ± 10.1 |
| 409 | Fusidic acid sodium | 93.0 ± 9.1 |  | 1049 | Indatraline hydrochloride | 103.0 ± 8.6 |
| 410 | Maprotiline hydrochloride | 93.0 ± 35.9 |  | 1050 | (±)-Octopamine hydrochloride | 103.0 ± 1.6 |
| 411 | Bezafibrate | 93.0 ± 7.9 |  | 1051 | SKF 86466 | 103.0 ± 12.5 |
| 412 | LY-310,762 hydrochloride | 93.0 ± 7.5 |  | 1052 | Iproniazid phosphate | 103.0 ± 5.6 |
| 413 | beta-Lapachone | 93.1 ± 5.0 |  | 1053 | (-)-Sulpiride | 103.0 ± 8.9 |
| 414 | (±)-Ibotenic acid | 93.1 ± 9.6 |  | 1054 | Carbachol | 103.1 ± 3.5 |
| 415 | Tyrphostin A9 | 93.2 ± 14.5 |  | 1055 | SR 2640 | 103.1 ± 17.1 |
| 416 | PK 11195 | 93.2 ± 6.1 |  | 1056 | DL-alpha-Difluoromethylornithine hydrochloride | 103.2 ± 12.1 |
| 417 | Cyclothiazide | 93.2 ± 4.7 |  | 1057 | ARL 67156 trisodium salt | 103.2 ± 16.2 |
| 418 | L-703,606 oxalate salt hydrate | 93.3 ± 4.4 |  | 1058 | (±)-PD 128,907 hydrochloride | 103.2 ± 2.1 |
| 419 | O-Phospho-L-serine | 93.4 ± 14.4 |  | 1059 | Ribavirin | 103.3 ± 14.0 |
| 420 | Ofloxacin | 93.4 ± 20.0 |  | 1060 | S(-)-Timolol maleate | 103.3 ± 4.5 |
| 421 | Quazinone | 93.4 ± 5.4 |  | 1061 | Phosphonoacetic acid | 103.4 ± 12.3 |
| 422 | ICI 63,137 | 93.4 ± 5.4 |  | 1062 | 4-(2-Aminoethyl)benzenesulfonyl fluoride hydrochloride | 103.5 ± 4.8 |
| 423 | Suramin sodium salt | 93.4 ± 9.0 |  | 1063 | (±)-alpha-Methyl-4-carboxyphenylglycine | 103.5 ± 10.0 |
| 424 | 6-Methoxy-1,2,3,4-tetrahydro-9H-pyrido[3,4b] indole | 93.4 ± 1.1 |  | 1064 | Moxisylyte hydrochloride | 103.5 ± 2.3 |
| 425 | (±)-Sotalol hydrochloride | 93.5 ± 12.0 |  | 1065 | YS-035 hydrochloride | 103.5 ± 12.5 |
| 426 | YM 976 | 93.5 ± 3.0 |  | 1066 | SR-95531 | 103.5 ± 3.4 |
| 427 | Meloxicam sodium | 93.6 ± 6.8 |  | 1067 | Methoctramine tetrahydrochloride | 103.6 ± 10.6 |
| 428 | SB 269970 hydrochloride | 93.6 ± 22.9 |  | 1068 | 1-(m-Chlorophenyl)-biguanide hydrochloride | 103.6 ± 13.0 |
| 429 | 4-Aminopyridine | 93.6 ± 7.2 |  | 1069 | (±)-Atenolol | 103.6 ± 10.0 |
| 430 | Meclofenamic acid sodium | 93.6 ± 9.6 |  | 1070 | 2',3'-dideoxycytidine | 103.6 ± 6.0 |
| 431 | Lamotrigine | 93.6 ± 9.2 |  | 1071 | 3-alpha,21-Dihydroxy-5-alpha-pregnan-20-one | 103.6 ± 6.1 |
| 432 | Retinoic acid | 93.6 ± 14.4 |  | 1072 | Nylidrin hydrochloride | 103.7 ± 6.3 |
| 433 | Beclomethasone | 93.7 ± 1.6 |  | 1073 | Dilazep hydrochloride | 103.7 ± 12.7 |
| 434 | LP 12 hydrochloride hydrate | 93.7 ± 9.5 |  | 1074 | Quinolinic acid | 103.7 ± 10.0 |
| 435 | TCPOBOP | 93.7 ± 8.4 |  | 1075 | Sulindac | 103.7 ± 16.6 |
| 436 | Nimodipine | 83.7 ± 4.5 |  | 1076 | R(-)-Isoproterenol (+)-bitartrate | 103.8 ± 21.4 |
| 437 | CB 1954 | 93.7 ± 2.8 |  | 1077 | LP44 | 103.8 ± 8.7 |
| 438 | Aurintricarboxylic acid | 93.7 ± 12.0 |  | 1078 | PHA-543613 | 103.8 ± 13.6 |
| 439 | Ketorolac tris salt | 93.7 ± 13.0 |  | 1079 | Phenytoin sodium | 103.8 ± 12.2 |
| 440 | Colchicine | 93.8 ± 5.1 |  | 1080 | 1-(5-Isoquinolinylsulfonyl)-2-methylpiperazine dihydrochloride | 103.8 ± 5.9 |
| 441 | 3-deazaadenosine | 93.8 ± 11.9 |  | 1081 | Na-p-Tosyl-L-lysine chloromethyl ketone hydrochloride | 103.9 ± 14.0 |
| 442 | McN-A-343 | 93.8 ± 10.4 |  | 1082 | Oxymetazoline hydrochloride | 103.9 ± 10.0 |
| 443 | Ketotifen fumarate | 93.8 ± 4.0 |  | 1083 | (+)-Pilocarpine hydrochloride | 103.9 ± 1.7 |
| 444 | BBMP | 93.9 ± 9.9 |  | 1084 | Tyrphostin 47 | 104.0 ± 4.7 |
| 445 | CP-66713 | 93.9 ± 10.2 |  | 1085 | 5-hydroxydecanoic acid sodium | 104.0 ± 7.6 |
| 446 | Azathioprine | 93.9 ± 5.0 |  | 1086 | L-Canavanine sulfate | 104.0 ± 8.6 |
| 447 | Guanidinyl-naltrindole di-trifluoroacetate | 93.9 ± 7.0 |  | 1087 | (+)-Cyclazocine | 104.1 ± 10.3 |
| 448 | Fexofenadine hydrochloride | 93.9 ± 3.6 |  | 1088 | Cyclosporin A | 104.1 ± 16.2 |
| 449 | 1-Phenyl-3-(2-thiazolyl)-2-thiourea | 94.0 ± 11.6 |  | 1089 | 2,4-Diamino-6-pyrimidinone | 104.1 ± 13.8 |
| 450 | Dihydro-beta-erythroidine hydrobromide | 94.0 ± 5.4 |  | 1090 | Alprenolol hydrochloride | 104.1 ± 15.8 |
| 451 | Cimetidine | 94.0 ± 4.0 |  | 1091 | Nemadipine-A | 104.1 ± 11.6 |
| 452 | Cortisone | 94.0 ± 5.3 |  | 1092 | (-)-MK-801 hydrogen maleate | 104.1 ± 10.2 |
| 453 | JS-K | 94.0 ± 5.4 |  | 1093 | Tamoxifen citrate | 104.2 ± 20.7 |
| 454 | CGP-74514A hydrochloride | 94.1 ± 9.9 |  | 1094 | U-69593 | 104.2 ± 10.1 |
| 455 | 5-(N-Ethyl-N-isopropyl)amiloride | 94.1 ± 7.3 |  | 1095 | GR 127935 hydrochloride hydrate | 104.2 ± 8.3 |
| 456 | Metergoline | 94.1 ± 8.5 |  | 1096 | Trimipramine maleate | 104.3 ± 7.1 |
| 457 | 6-Hydroxymelatonin | 94.1 ± 9.8 |  | 1097 | L-alpha-Methyl-p-tyrosine | 104.3 ± 4.3 |
| 458 | Chloroquine diphosphate | 94.1 ± 9.1 |  | 1098 | Pirenzepine dihydrochloride | 104.3 ± 2.0 |
| 459 | (±)-p-Aminoglutethimide | 94.1 ± 8.7 |  | 1099 | GR-89696 fumarate | 104.3 ± 9.0 |
| 460 | BMS-193885 | 94.2 ± 1.0 |  | 1100 | 2-methoxyestradiol | 104.4 ± 10.2 |
| 461 | Cefotaxime sodium | 94.2 ± 6.2 |  | 1101 | Desipramine hydrochloride | 104.4 ± 9.0 |
| 462 | Loperamide hydrochloride | 94.2 ± 11.6 |  | 1102 | Harmane | 104.5 ± 8.0 |
| 463 | Org 24598 lithium salt | 94.3 ± 5.8 |  | 1103 | Carbetapentane citrate | 104.5 ± 6.7 |
| 464 | N-(2-[4-(4-Chlorophenyl)piperazin-1-yl]ethyl)-3-methoxybenzamide | 94.3 ± 6.0 |  | 1104 | Hemicholinium-3 | 104.5 ± 15.9 |
| 465 | Bumetanide | 94.3 ± 2.5 |  | 1105 | Caroverine hydrochloride | 104.5 ± 21.3 |
| 466 | BTCP hydrochloride | 94.3 ± 14.0 |  | 1106 | Procaine hydrochloride | 104.5 ± 8.1 |
| 467 | (+)-Catechin Hydrate | 94.3 ± 10.9 |  | 1107 | Phenylbutazone | 104.5 ± 10.1 |
| 468 | Trovafloxacin mesylate | 94.4 ± 17.2 |  | 1108 | Bay 11-7082 | 104.6 ± 21.3 |
| 469 | Lumefantrine | 94.4 ± 10.9 |  | 1109 | Cephalothin sodium | 104.6 ± 12.0 |
| 470 | GW9508 | 94.4 ± 0.8 |  | 1110 | Amantadine hydrochloride | 104.6 ± 3.5 |
| 471 | Clemastine fumarate | 94.4 ± 2.5 |  | 1111 | ICI 204,448 hydrochloride | 104.6 ± 7.5 |
| 472 | NBQX disodium | 94.5 ± 3.8 |  | 1112 | Trazodone hydrochloride | 104.7 ± 11.3 |
| 473 | Fluspirilene | 94.5 ± 10.0 |  | 1113 | 2-Methyl-5-hydroxytryptamine maleate | 104.7 ± 14.7 |
| 474 | Spironolactone | 94.5 ± 9.9 |  | 1114 | 17alpha-hydroxyprogesterone | 104.8 ± 10.6 |
| 475 | SB 216763 | 94.5 ± 10.2 |  | 1115 | (+)-MK-801 hydrogen maleate | 104.8 ± 18.5 |
| 476 | 2-Cyclooctyl-2-hydroxyethylamine hydrochloride | 94.5 ± 6.6 |  | 1116 | Famciclovir | 104.8 ± 9.2 |
| 477 | Lonidamine | 94.6 ± 7.2 |  | 1117 | Hexahydro-sila-difenidol hydrochloride, p-fluoro analog | 104.8 ± 7.7 |
| 478 | (±) trans-U-50488 methanesulfonate | 94.6 ± 5.3 |  | 1118 | Alaproclate hydrochloride | 104.8 ± 8.1 |
| 479 | Hypotaurine | 94.6 ± 26.1 |  | 1119 | SC 19220 | 105.0 ± 9.4 |
| 480 | LY-294,002 hydrochloride | 94.6 ± 11.0 |  | 1120 | DM 235 | 105.1 ± 10.7 |
| 481 | Amifostine | 94.6 ± 2.0 |  | 1121 | Pinacidil | 105.1 ± 8.3 |
| 482 | Isoxanthopterin | 94.7 ± 15.0 |  | 1122 | 2,2'-Bipyridyl | 105.2 ± 7.5 |
| 483 | CNQX disodium | 94.7 ± 15.8 |  | 1123 | U-62066 | 105.2 ± 12.6 |
| 484 | Tetraethylammonium chloride | 94.7 ± 8.6 |  | 1124 | Naphazoline hydrochloride | 105.3 ± 13.5 |
| 485 | Cambinol | 94.8 ± 8.3 |  | 1125 | 4-Hydroxybenzhydrazide | 105.3 ± 8.4 |
| 486 | SID7969543 | 94.8 ± 24.3 |  | 1126 | Linopirdine | 105.3 ± 3.6 |
| 487 | 3,7-Dimethyl-1-propargylxanthine | 94.8 ± 1.8 |  | 1127 | PAC-1 | 105.3 ± 4.5 |
| 488 | SR 59230A oxalate | 94.8 ± 26.7 |  | 1128 | Cirazoline hydrochloride | 105.5 ± 7.7 |
| 489 | Dantrolene sodium | 94.8 ± 9.5 |  | 1129 | Adenosine 3',5'-cyclic monophosphate | 105.5 ± 3.5 |
| 490 | DFB | 94.8 ± 1.4 |  | 1130 | L-745,870 hydrochloride | 105.7 ± 4.1 |
| 491 | SNC80 | 94.8 ± 9.3 |  | 1131 | Rhodblock 6 | 105.7 ± 10.7 |
| 492 | (±)-Muscarine chloride | 94.8 ± 15.8 |  | 1132 | Quinelorane dihydrochloride | 105.7 ± 9.9 |
| 493 | Paliperidone | 94.9 ± 1.6 |  | 1133 | Cilostazol | 105.8 ± 41.7 |
| 494 | NS-1619 | 94.9 ± 11.8 |  | 1134 | Spermine tetrahydrochloride | 105.8 ± 16.0 |
| 495 | (±)-p-Chlorophenylalanine | 94.9 ± 9.4 |  | 1135 | ML 10302 | 105.8 ± 6.4 |
| 496 | Tyrphostin 51 | 94.9 ± 2.9 |  | 1136 | (-)-Eseroline fumarate | 105.8 ± 9.3 |
| 497 | 4-Hydroxy-3-methoxyphenylacetic acid | 94.9 ± 9.6 |  | 1137 | Levallorphan tartrate | 105.9 ± 7.3 |
| 498 | Apomorphine hydrochloride hemihydrate | 95.0 ± 6.4 |  | 1138 | 5,5-Dimethyl-1-pyrroline-N-oxide | 106.0 ± 5.3 |
| 499 | Betaine aldehyde chloride | 95.0 ± 5.8 |  | 1139 | Gemcitabine hydrochloride | 106.0 ± 4.9 |
| 500 | D-Cycloserine | 94.5 ± 6.4 |  | 1140 | (±)-Propranolol hydrochloride | 106.0 ± 10.5 |
| 501 | Ivermectin | 94.5 ± 8.2 |  | 1141 | Vincristine sulfate | 106.1 ± 21.2 |
| 502 | TMB-8 hydrochloride | 95.0 ± 0.4 |  | 1142 | Nortriptyline hydrochloride | 106.1 ± 17.3 |
| 503 | MHPG piperazine | 95.1 ± 7.6 |  | 1143 | Nalidixic acid sodium | 106.1 ± 13.7 |
| 504 | Idarubicin | 95.1 ± 1.7 |  | 1144 | PPADS | 106.1 ± 11.3 |
| 505 | Bromoacetylcholine bromide | 95.1 ± 8.8 |  | 1145 | Putrescine dihydrochloride | 106.2 ± 11.7 |
| 506 | S-(4-Nitrobenzyl)-6-thioinosine | 95.1 ± 33.3 |  | 1146 | Haloperidol | 106.2 ± 9.5 |
| 507 | SB 205384 | 95.1 ± 6.2 |  | 1147 | Paromomycin sulfate | 106.3 ± 7.8 |
| 508 | TMPH hydrochloride | 95.1 ± 13.7 |  | 1148 | Pentolinium di[L(+)-tartrate] | 106.3 ± 2.7 |
| 509 | Tetraisopropyl pyrophosphoramide | 952 ± 1.5 |  | 1149 | Xylazine hydrochloride | 106.3 ± 5.8 |
| 510 | N-Phenylanthranilic acid | 95.2 ± 6.4 |  | 1150 | CGP 20712A methanesulfonate | 106.4 ± 13.7 |
| 511 | Nimustine hydrochloride | 95.2 ± 13.6 |  | 1151 | (+)-Quisqualic acid | 106.4 ± 10.3 |
| 512 | Cibenzoline succinate | 95.2 ± 7.2 |  | 1152 | Decamethonium dibromide | 106.4 ± 21.3 |
| 513 | Aconitine | 95.2 ± 4.4 |  | 1153 | H-8 dihydrochloride | 106.4 ± 6.7 |
| 514 | BP 897 | 95.2 ± 9.6 |  | 1154 | Metoclopramide hydrochloride | 106.5 ± 2.9 |
| 515 | Efaroxan hydrochloride | 95.2 ± 6.3 |  | 1155 | (-)-Cotinine | 106.5 ± 4.3 |
| 516 | Bay 11-7085 | 95.2 ± 7.7 |  | 1156 | L-Mimosine from Koa hoale seeds | 106.5 ± 13.7 |
| 517 | SC-51089 hydrate | 95.2 ± 13.2 |  | 1157 | Melatonin | 106.6 ± 11.6 |
| 518 | Benzamil hydrochloride | 95.3 ± 5.6 |  | 1158 | S(-)-UH-301 hydrochloride | 106.6 ± 12.1 |
| 519 | (±)-Isoproterenol hydrochloride | 95.3 ± 10.4 |  | 1159 | Ipratropium bromide | 106.7 ± 26.2 |
| 520 | (±)-Bay K 8644 | 95.3 ± 3.4 |  | 1160 | Xylometazoline hydrochloride | 106.7 ± 10.4 |
| 521 | SKF-525A hydrochloride | 95.3 ± 9.0 |  | 1161 | Taurine | 106.8 ± 10.4 |
| 522 | Triamterene | 95.3 ± 10.4 |  | 1162 | Prilocaine hydrochloride | 106.9 ± 9.7 |
| 523 | 1-(5-Isoquinolinylsulfonyl)-3-methylpiperazine dihydrochloride | 95.4 ± 14.7 |  | 1163 | Naltriben methanesulfonate | 106.9 ± 8.0 |
| 524 | 4-Amino-1,8-naphthalimide | 95.4 ± 12.1 |  | 1164 | MG 624 | 106.9 ± 11.5 |
| 525 | Pentylenetetrazole | 95.4 ± 8.7 |  | 1165 | Ancitabine hydrochloride | 106.9 ± 5.2 |
| 526 | 5-fluoro-5'-deoxyuridine | 95.4 ± 4.3 |  | 1166 | Bisoprolol hemifumarate salt | 106.9 ± 6.1 |
| 527 | Ifenprodil tartrate | 95.4 ± 10.6 |  | 1167 | Telenzepine dihydrochloride | 107.0 ± 15.2 |
| 528 | Ruthenium red | 95.4 ± 2.3 |  | 1168 | Proglumide | 107.1 ± 0.6 |
| 529 | R(+)-IAA-94 | 95.5 ± 13.0 |  | 1169 | L-Methionine sulfoximine | 107.1 ± 5.4 |
| 530 | (±)-Normetanephrine hydrochloride | 95.5 ± 24.8 |  | 1170 | Mevastatin | 107.1 ± 10.0 |
| 531 | D-609 potassium | 95.5 ± 4.1 |  | 1171 | Ro 8-4304 | 107.2 ± 11.5 |
| 532 | A3 hydrochloride | 95.5 ± 8.6 |  | 1172 | Phaclofen | 107.2 ± 18.9 |
| 533 | 5-(N,N-Dimethyl)amiloride hydrochloride | 95.5 ± 2.7 |  | 1173 | Tizanidine hydrochloride | 107.2 ± 6.1 |
| 534 | Propantheline bromide | 95.5 ± 4.8 |  | 1174 | O-Methylserotonin hydrochloride | 107.2 ± 4.7 |
| 535 | Ibandronate sodium | 95.5 ± 10.4 |  | 1175 | Stattic | 107.3 ± 19.6 |
| 536 | CX 546 | 95.5 ± 26.6 |  | 1176 | Doxylamine succinate | 107.4 ± 0.7 |
| 537 | Tetramisole hydrochloride | 95.5 ± 22.8 |  | 1177 | Ropinirole hydrochloride | 107.5 ± 15.4 |
| 538 | GABA | 95.6 ± 8.2 |  | 1178 | Muscimol hydrobromide | 107.5 ± 2.4 |
| 539 | Cephradine | 95.6 ± 1.1 |  | 1179 | Mibefradil dihydrochloride | 107.6 ± 26.6 |
| 540 | Enalaprilat dihydrate | 95.6 ± 2.1 |  | 1180 | Hydroxyurea | 107.6 ± 23.3 |
| 541 | 3-Tropanylindole-3-carboxylate methiodide | 95.6 ± 4.9 |  | 1181 | S(+)-Ibuprofen | 107.7 ± 17.0 |
| 542 | Cyproterone acetate | 95.6 ± 2.1 |  | 1182 | (2S,1'S,2'S)-2-(carboxycyclopropyl)glycine | 107.7 ± 15.6 |
| 543 | PAPP | 95.6 ± 9.9 |  | 1183 | Chlorothiazide | 107.7 ± 8.4 |
| 544 | A-68930 hydrochloride | 95.6 ± 9.3 |  | 1184 | VER-3323 hemifumarate salt | 107.7 ± 7.9 |
| 545 | Hydrochlorothiazide | 95.6 ± 3.9 |  | 1185 | 6-Nitroso-1,2-benzopyrone | 107.9 ± 12.7 |
| 546 | DL-p-Chlorophenylalanine methyl ester hydrochloride | 95.7 ± 6.7 |  | 1186 | PD-166285 hydrate | 108.1 ± 4.5 |
| 547 | GBR-12909 dihydrochloride | 95.7 ± 6.3 |  | 1187 | Ethopropazine hydrochloride | 108.3 ± 15.2 |
| 548 | Acetylthiocholine chloride | 95.7 ± 4.8 |  | 1188 | 1-Amino-1-cyclohexanecarboxylic acid hydrochloride | 108.3 ± 1.1 |
| 549 | Furosemide | 95.7 ± 5.9 |  | 1189 | S(-)-3PPP hydrochloride | 108.4 ± 3.1 |
| 550 | Tranilast | 95.7 ± 1.4 |  | 1190 | MRS 2179 | 108.4 ± 2.3 |
| 551 | (±)-Epinephrine hydrochloride | 95.7 ± 7.0 |  | 1191 | Norcantharidin | 108.5 ± 19.2 |
| 552 | IMS2186 | 95.7 ± 10.2 |  | 1192 | L-687,384 hydrochloride | 108.6 ± 4.6 |
| 553 | Benoxathian hydrochloride | 95.8 ± 4.4 |  | 1193 | Hydroxytacrine maleate | 108.6 ± 13.0 |
| 554 | 3,4-Dichloroisocoumarin | 95.8 ± 9.3 |  | 1194 | N2-Ethyl-2'-deoxyguanosine | 108.7 ± 6.3 |
| 555 | Caffeine | 95.8 ± 4.7 |  | 1195 | (±)-Thalidomide | 108.7 ± 11.6 |
| 556 | Serotonin hydrochloride | 95.8 ± 9.3 |  | 1196 | Ro 20-1724 | 108.8 ± 2.1 |
| 557 | 6-Methyl-2-(phenylethynyl)pyridine hydrochloride | 95.8 ± 6.2 |  | 1197 | (-)-trans-(1S,2S)-U-50488 hydrochloride | 108.9 ± 5.7 |
| 558 | (+)-Hydrastine | 95.9 ± 10.1 |  | 1198 | alpha-Methyl-DL-tyrosine methyl ester hydrochloride | 108.9 ± 8.2 |
| 559 | L-Beta-threo-benzyl-aspartate | 95.9 ± 9.2 |  | 1199 | 6-Aminohexanoic acid | 109.0 ± 9.6 |
| 560 | Aniracetam | 95.9 ± 3.1 |  | 1200 | Picotamide | 109.0 ± 15.4 |
| 561 | SKF 89976A hydrochloride | 95.9 ± 25.1 |  | 1201 | NG-Nitro-L-arginine methyl ester hydrochloride | 109.0 ± 16.7 |
| 562 | N,N,N',N'-Tetramethylazodicarboxamide | 95.9 ± 2.2 |  | 1202 | CR 2249 | 109.1 ± 19.6 |
| 563 | Ro 25-6981 hydrochloride | 95.9 ± 5.0 |  | 1203 | Tolazamide | 109.2 ± 9.0 |
| 564 | Steviol | 95.9 ± 19.9 |  | 1204 | Prazosin hydrochloride | 109.3 ± 23.7 |
| 565 | Triamcinolone | 95.9 ± 21.3 |  | 1205 | Zaprinast | 109.4 ± 6.4 |
| 566 | 5,5-Diphenylhydantoin | 95.9 ± 12.2 |  | 1206 | D(-)-2-Amino-5-phosphonopentanoic acid | 109.4 ± 11.9 |
| 567 | Arecaidine propargyl ester hydrobromide | 96.0 ± 12.2 |  | 1207 | N-Methyl-D-aspartic acid | 109.5 ± 4.3 |
| 568 | Benztropine mesylate | 96.0 ± 5.8 |  | 1208 | 3-Tropanyl-indole-3-carboxylate hydrochloride | 109.6 ± 9.2 |
| 569 | Clorgyline hydrochloride | 96.0 ± 4.2 |  | 1209 | NNC 55-0396 | 109.8 ± 31.5 |
| 570 | MDL 28170 | 96.0 ± 2.8 |  | 1210 | 2,6-Difluoro-4-[2-(phenylsulfonylamino)ethylthio]phenoxyacetamide | 109.8 ± 13.1 |
| 571 | Cyproheptadine hydrochloride | 96.0 ± 5.5 |  | 1211 | BNTX maleate salt hydrate | 109.9 ± 5.7 |
| 572 | Riluzole | 96.0 ± 3.6 |  | 1212 | Memantine hydrochloride | 109.9 ± 10.5 |
| 573 | (±)-2-Amino-3-phosphonopropionic acid | 96.0 ± 3.9 |  | 1213 | 2,3-Butanedione monoxime | 109.9 ± 13.3 |
| 574 | Propofol | 96.0 ± 3.0 |  | 1214 | Piracetam | 110.0 ± 4.3 |
| 575 | 8-Cyclopentyl-1,3-dimethylxanthine | 96.1 ± 9.6 |  | 1215 | Doxepin hydrochloride | 110.0 ± 13.2 |
| 576 | Acetamide | 96.1 ± 7.6 |  | 1216 | Chelidamic acid | 110.2 ± 8.8 |
| 577 | Arcaine sulfate | 96.1 ± 6.4 |  | 1217 | BIA 2-093 | 110.6 ± 5.0 |
| 578 | Nitrendipine | 96.1 ± 17.4 |  | 1218 | Cortexolone | 110.6 ± 19.2 |
| 579 | R(-)-Propylnorapomorphine hydrochloride | 96.1 ± 7.1 |  | 1219 | T0070907 | 110.9 ± 6.0 |
| 580 | Voriconazole | 96.1 ± 10.4 |  | 1220 | R-(+)-7-Hydroxy-DPAT hydrobromide | 110.9 ± 7.0 |
| 581 | Primidone | 96.1 ± 4.9 |  | 1221 | Naltrindole hydrochloride | 111.0 ± 9.1 |
| 582 | CP-135807 | 96.1 ± 6.8 |  | 1222 | (±)-Taxifolin | 111.1 ± 7.5 |
| 583 | N-Methyldopamine hydrochloride | 96.1 ± 15.9 |  | 1223 | Propafenone hydrochloride | 111.3 ± 10.1 |
| 584 | (±)-AMPA hydrobromide | 96.2 ± 10.6 |  | 1224 | 3-Nitropropionic acid | 111.3 ± 13.2 |
| 585 | JL-18 | 96.2 ± 7.5 |  | 1225 | Methapyrilene hydrochloride | 111.4 ± 10.2 |
| 586 | Lidocaine N-ethyl bromide quaternary salt | 96.2 ± 12.5 |  | 1226 | Sobuzoxane | 111.5 ± 7.6 |
| 587 | Phenylbenzene-omega-phosphono-alpha-amino acid | 96.2 ± 4.8 |  | 1227 | Quinidine sulfate | 111.6 ± 16.6 |
| 588 | 1-Phenylbiguanide | 96.2 ± 2.3 |  | 1228 | N-omega-Methyl-5-hydroxytryptamine oxalate salt | 111.6 ± 33.8 |
| 589 | R(+)-SCH-23390 hydrochloride | 96.2 ± 12.3 |  | 1229 | CP-31398 dihydrochloride hydrate | 111.8 ± 1.4 |
| 590 | Ganciclovir | 96.3 ± 6.5 |  | 1230 | NADPH tetrasodium | 112.0 ± 13.1 |
| 591 | NSC 95397 | 96.3 ± 15.0 |  | 1231 | S-Methylisothiourea hemisulfate | 112.2 ± 21.2 |
| 592 | Glipizide | 96.3 ± 1.2 |  | 1232 | Methiothepin mesylate | 112.2 ± 9.4 |
| 593 | Cefazolin sodium | 96.3 ± 4.6 |  | 1233 | NG-Monomethyl-L-arginine acetate | 112.2 ± 7.1 |
| 594 | Nicardipine hydrochloride | 96.3 ± 24.2 |  | 1234 | BRL 52537 hydrochloride | 112.4 ± 4.2 |
| 595 | Droxinostat | 96.3 ± 1.9 |  | 1235 | Spiroxatrine | 112.7 ± 3.0 |
| 596 | Genipin | 96.3 ± 9.0 |  | 1236 | Idazoxan hydrochloride | 112.7 ± 7.0 |
| 597 | L-N6-(1-Iminoethyl)lysine hydrochloride | 96.4 ± 1.7 |  | 1237 | Metolazone | 112.9 ± 23.7 |
| 598 | Sorbinil | 96.4 ± 14.0 |  | 1238 | (±)-Vesamicol hydrochloride | 112.9 ± 7.4 |
| 599 | Pirfenidone | 96.4 ± 15.8 |  | 1239 | (-)-Tetramisole hydrochloride | 112.9 ± 6.8 |
| 600 | Sodium Oxamate | 96.5 ± 14.4 |  | 1240 | L-Glutamic acid hydrochloride | 113.0 ± 19.3 |
| 601 | NO-711 hydrochloride | 95.5 ± 5.2 |  | 1241 | Niflumic acid | 113.3 ± 19.3 |
| 602 | Rauwolscine hydrochloride | 96.5 ± 8.7 |  | 1242 | 3-Morpholinosydnonimine hydrochloride | 114.0 ± 15.4 |
| 603 | cis-(Z)-Flupenthixol dihydrochloride | 96.5 ± 4.1 |  | 1243 | (±)-Verapamil hydrochloride | 114.0 ± 7.1 |
| 604 | 3-Amino-1-propanesulfonic acid sodium | 96.5 ± 4.6 |  | 1244 | Nimesulide | 114.0 ± 22.1 |
| 605 | SC-58125 | 96.5 ± 13.6 |  | 1245 | (±)-CGP-12177A hydrochloride | 114.1 ± 10.3 |
| 606 | Sivelestat sodium salt hydrate | 96.6 ± 6.4 |  | 1246 | Naloxone hydrochloride | 114.3 ± 14.2 |
| 607 | Epinastine hydrochloride | 96.6 ± 14.2 |  | 1247 | GW9662 | 114.5 ± 11.5 |
| 608 | Cystamine dihydrochloride | 96.6 ± 12.8 |  | 1248 | Noscapine hydrchloride | 114.6 ± 20.4 |
| 609 | Chlorprothixene hydrochloride | 96.6 ± 7.7 |  | 1249 | 1-(2-Methoxyphenyl)piperazine hydrochloride | 114.8 ± 8.3 |
| 610 | (±)-HA-966 | 96.6 ± 6.4 |  | 1250 | alpha-Methyl-5-hydroxytryptamine maleate | 115.5 ± 12.2 |
| 611 | ATPA | 96.6 ± 6.2 |  | 1251 | ZM 39923 hydrochloride | 115.5 ± 23.0 |
| 612 | SD-169 | 96.7 ± 5.5 |  | 1252 | 1-Methylimidazole | 115.7 ± 16.4 |
| 613 | Minoxidil | 96.7 ± 6.2 |  | 1253 | (-)-Perillic acid | 115.8 ± 16.6 |
| 614 | Promethazine hydrochloride | 96.7 ± 11.4 |  | 1254 | Quinine sulfate | 116.2 ± 16.5 |
| 615 | Imipenem monohydrate | 96.8 ± 2.2 |  | 1255 | p-MPPF dihydrochloride | 117.8 ± 19.4 |
| 616 | Piperaquine tetraphosphate tetrahydrate | 96.8 ± 7.2 |  | 1256 | SKF 95282 dimaleate | 120.1 ± 40.0 |
| 617 | 3-Iodo-L-tyrosine | 96.8 ± 8.8 |  | 1257 | 5-Nitro-2-(3-phenylpropylamino)benzoic acid | 120.8 ± 13.5 |
| 618 | Aprindine hydrochloride | 96.8 ± 13.3 |  | 1258 | NG-Nitro-L-arginine | 120.0 ± 6.8 |
| 619 | S-(-)-Eticlopride hydrochloride | 96.8 ± 1.1 |  | 1259 | MDL 26,630 trihydrochloride | 124.5 ± 16.5 |
| 620 | (+)-Chlorpheniramine maleate | 96.8 ± 6.2 |  | 1260 | 7-Nitroindazole | 126.0 ± 15.1 |
| 621 | Astaxanthin | 96.8 ± 4.6 |  | 1261 | S-Nitroso-N-acetylpenicillamine | 127.5 ± 10.1 |
| 622 | Ara-G hydrate | 96.8 ± 6.9 |  | 1262 | Methoxamine hydrochloride | 132.3 ± 57.0 |
| 623 | Picrotoxin | 96.8 ± 4.1 |  | 1263 | JFD00244 | 113.9 ± 48.7 |
| 624 | Nialamide | 96.8 ± 7.4 |  | 1264 | (±)-Butaclamol hydrochloride | ND |
| 625 | Lomefloxacin hydrochloride | 96.9 ± 10.1 |  | 1265 | (±)-Quinpirole dihydrochloride | ND |
| 626 | Eletriptan hydrobromide | 96.9 ± 5.3 |  | 1266 | Aurothioglucose | ND |
| 627 | nor-Binaltorphimine dihydrochloride | 96.9 ± 14.7 |  | 1267 | Bethanechol chloride | ND |
| 628 | Bicalutamide (CDX) | 96.9 ± 5.3 |  | 1268 | DL-Buthionine-[S,R]-sulfoximine | ND |
| 629 | Cinoxacin | 96.9 ± 2.7 |  | 1269 | GBR-12935 dihydrochloride | ND |
| 630 | (±)-gamma-Vinyl GABA | 96.9 ± 7.5 |  | 1270 | Guanfacine hydrochloride | ND |
| 631 | 3-Tropanyl-3,5-dichlorobenzoate | 96.9 ± 4.1 |  | 1271 | GW5074 | ND |
| 632 | DL-threo-beta-hydroxyaspartic acid | 97.0 ± 9.5 |  | 1272 | L-162,313 | ND |
| 633 | 3,4-Dihydroxyphenylacetic acid | 97.0 ± 9.9 |  | 1273 | m-Iodobenzylguanidine hemisulfate | ND |
| 634 | Olomoucine | 97.0 ± 10.1 |  | 1274 | MK-912 | ND |
| 635 | Milrinone | 97.0 ± 4.9 |  | 1275 | PD-407824 | ND |
| 636 | Antozoline hydrochloride | 97.0 ± 6.0 |  | 1276 | Progesterone | ND |
| 637 | S15535 | 97.0 ± 1.3 |  | 1277 | Propentofylline | ND |
| 638 | Urapidil hydrochloride | 97.1 ± 1.5 |  | 1278 | Protoporphyrin IX disodium | ND |
| 639 | Trifluoperazine dihydrochloride | 97.1 ± 25.1 |  |  |  |  |
| 640 | L-Arginine | 97.1 ± 5.5 |  |  |  |  |
